# Supplementary material for: De novo design of luciferases using deep learning
Source: Nature. 2023 Feb 22;614(7949):774–80. doi: 10.1038/s41586-023-05696-3 (PMC9946828; doi:10.1038/s41586-023-05696-3)
Supplement: Supplementary file 1 — This file contains the Methods section [file 41586_2023_5696_MOESM1_ESM.pdf]

nature portfolio

https://doi.org/10.1038/s41586-023-05696-3

Supplementary information

De novo design of luciferases using deep learning

In the format provided by the authors and unedited

Supplementary information for "De novo design of luciferases using deep learning"

Authors: Andy Hsien-Wei Yeh[1,2,3†*], Christoffer Norn[1,2†], Yakov Kipnis[1,2,6], Doug Tischer[1,2], Samuel J. Pellock[1,2], Declan Evans[4], Pengchen Ma[4,5], Gyu Rie Lee[1,2], Jason Z. Zhang[1,2], Ivan Anishchenko[1,2], Brian Coventry[1,2,6], Longxing Cao[1,2], Justas Dauparas[1,2], Samer Halabiya[2], Michelle DeWitt[2], Lauren Carter[2], K. N. Houk[4] & David Baker[1,2,6*]

Affiliations:
[1]Department of Biochemistry, University of Washington, Seattle, Washington, USA
[2]Institute for Protein Design, University of Washington, Seattle, Washington, USA
[3]Department of Biomolecular Engineering, University of California, Santa Cruz, CA, USA
[4]Department of Chemistry and Biochemistry, University of California, Los Angeles, CA, USA
[5]School of Chemistry, Xi'an Key Laboratory of Sustainable Energy Materials Chemistry, MOE Key Laboratory for Nonequilibrium Synthesis and Modulation of Condensed Matter, Xi'an Jiaotong University, Xi'an, China
[6]Howard Hughes Medical Institute, University of Washington, Seattle, Washington, USA

[†]These authors contributed equally
*Corresponding authors. Email: hsyeh@ucsc.edu or dabaker@uw.edu

Methods:

1. Materials and general methods

Synthetic genes and oligonucleotides were purchased from Integrated DNA Technologies or GenScript. The synthetic gene was inserted into modified pET29b+ vectors, containing N- or C-terminal hexahistidine tag. Restriction endonucleases, Q5 PCR polymerase, USER enzyme, NEBNext end repair module, and T4 ligase were purchased from NEB. Plasmid DNA, PCR products, or digested fragments were purified by Qiagen DNA purification kits. DNA sequences were analyzed by Genewiz. Coelenterazine (CTZ) was purchased from Gold Biotechnology. Diphenylterazine (DTZ), pyridyl diphenylterazine (8pyDTZ), and Furimazine (FRZ) were purchased from MedChemExpress. All other coelenterazine analogs (bis-CTZ: bisdeoxycoelenterazine; f-CTZ: f-Coelenterazine; e-CTZ: e-Coelenterazine-F; PP-CTZ: methoxy e-Coelenterazine; v-CTZ: v-Coelenterazine; h-CTZ: 2-deoxycoelenterazine) were ordered from NanoLight. All other chemicals were purchased from Sigma-Aldrich or Fisher Scientific and used without further purification. To identify the molecular mass of each protein, intact mass spectra were obtained via reverse-phase LC/MS on an Agilent 6230B TOF on an AdvanceBio RP-Desalting column and subsequently deconvoluted by Bioconfirm software (10.0) using a total entropy algorithm. ÄKTA pure M with UNICORN 6.3.2 Workstation control (GE Healthcare) coupled with a Superdex 75 Increase 10/300 GL column was used for size exclusion chromatography. DNA and protein concentrations were determined by an 8-channel NanoDrop UV/vis spectrometer. CD spectra and CD melting experiments were performed by default setting on a J-1500 Circular Dichroism Spectropolarimeter (Jasco). All luminescence measurements were acquired by a Biotek Synergy Neo2 Multi-Mode Plate Reader. To convert relative arbitrary unit (RLU) to the number of photons, Neo2 plate reader was calibrated by determining the chemiluminescence of luminol with known quantum yield in the presence of horseradish peroxidase and hydrogen peroxide in K_2CO_3 aqueous solution as previously described[42]. SDS PAGE and luminescence images were captured by a Bio-Rad ChemiDoc XRS+. HEK293T (CRL-11268) and HeLa (CCL-2) cells were obtained from ATCC. Images were analyzed using the Fiji image analysis software.

2. General procedures for protein production and purification

Lemo21(DE3) strain was used for transformation with the pET29b+ plasmid encoding the gene of interest. Transformed cells were grown for 12 h in LB medium supplemented with kanamycin. Cells were inoculated at 1:50 ratio in 100 mL fresh TB medium, grown at 37 °C for 4 h, and then induced by IPTG for an additional 18 h at 16 °C. Cells were harvested by centrifugation at 4,000g for 10 min and resuspended in 30 mL lysis buffer (20 mM Tris-HCl pH 8.0, 300 mM NaCl, 30 mM imidazole, and Pierce™ Protease Inhibitor Tablets). Cell resuspensions were lysed by sonication for 5 min (10 s per cycle). Lysates were clarified by centrifugation at 24,000g at 12 °C for 40 min and pre-equilibrated with 1 mL of Ni-NTA nickel agarose at 4 °C for 1 h. The resin was washed twice with 10 mL wash buffer and then eluted in 1 mL elution buffer (20 mM Tris-HCl pH 8.0, 300 mM NaCl, 300 mM imidazole). The eluted proteins were purified by size exclusion chromatography in PBS. Fractions were collected based on A280 trace, snap-frozen in liquid nitrogen, and stored at −80 °C.

3. Computational design of idealized scaffolds

Our generation of idealized NTF2-scaffolds can be divided into four parts: (3.1) Generation of seed-structures, (3.2) optimization of backbone geometries using trRosetta-based hallucination, (3.3) generation of structure-conditioned sequence models to bias design, (3.4) design and filtering.

3.1 Generation of seed structures

We thought to increase the set of NTF2 structures by complementing experimentally resolved structures from the PDB with highly accurate models generated by trRosetta[20]. To achieve this, we first collected 85 NTF2-like protein structures from the PDB based on SCOPe annotation (d. 17.4 SCOPe v2.05). Corresponding sequences were then used as queries to collect sequence homologs from UniProt by performing 8 iterations of hhblits at 1e-20 e-value cutoff against uniclust30_2018_08 database; default filtering cutoffs were relieved (-maxfilt 100000000 -neffmax 20 -nodiff -realign_max 10000000) to maximize the number of the output hits. All the hits were redundancy reduced using cd-hit[43] with a sequence identity cutoff of 60% yielding a set of 7,573 candidates for modeling.

To generate inputs for structure modeling with trRosetta, we built multiple sequence alignments (MSAs) for each of the 7,573 selected sequences with *hhblits* using a more conservative e-value cutoff of 1e-50; the resulting MSAs were also complemented by hits from *hmmsearch* against uniref100 (release-2019_11) with the bit-score threshold of 115 (i.e. ~1bit per position). After joining the above two sets of alignments and filtering them at 90% sequence identity and 75% coverage cutoffs, only sequences with more than 50 homologs in the corresponding MSAs were retained for modeling (2,005 sequences). The filtered MSAs along with information on the top 25 putative structural homologs as identified by *hhsearch* against the PDB100 database of templates were used as inputs to the template-aware version of trRosetta[44] to predict residue pair distances and orientations. Network predictions were then used to reconstruct full atom 3D structure models using a Rosetta-based folding protocol described previously[20].

3.2 Hallucination of idealized NTF2s

Seeking to idealize the native structure seeds, we reasoned that trRosetta, a convolutional residual neural network, which predicts residue-residue orientations and distances from sequence, could serve as a key component in a protein idealizer. Previously, this network has been used to generate diverse proteins that resemble the “ideal” structures of de novo designed proteins by changing the protein sequence to optimize the contrast (KL-divergence) between the predicted geometry and that of randomly generated sequences[17].

For our purpose, the desired fold-space is not diverse but instead focused on the NTF2-like topology. To guarantee generation of ideal structures within this fold-space, we implemented a new fold-specific loss-function, which biased hallucinations based on observed geometries in native crystal structures. As many experimentally characterized NTF2s contain non-ideal regions, we began by creating a set (χ) of trimmed but ideal NTF2s by manually removing non-ideal structural elements such as kinked helices, and long or rarely observed loops. For each seed structure, we then used a structure-based sequence alignment method (see 3.3) to find equivalent positions between the seed structure and χ. Residue pairs were considered to be in a conserved tertiary motif (TERM) if there were 5 or more equivalent positions in χ. The smooth probability distributions based on observed geometries in χ were then computed. For distances we used a Gaussian distribution with mean equal to the true distance denoted by D and standard deviation denoted by σ equal to 0.5 Å. The probability density function for distances d is given by:

$$f(d;\ D,\sigma) = \frac{1}{\sqrt{2\pi\sigma^2}\exp\left(-\frac{(d-D)^2}{2\sigma^2}\right)}$$

Using this density function one can construct a categorical distribution for binned distances by evaluating this function at the centers of the bins and then normalizing by a sum of all values in different bins. Similarly, a von Mises distribution was used for omega angle smoothing with probability density function given by $f(\omega;\ \Omega,\kappa) = N(\kappa)\exp[\kappa\cos(\omega-\Omega)]$ where $N(\kappa)$ is a

normalizing constant, Ω is the crystal value, κ is the inverse variance chosen to be 100, and ω is the smoothed angle. For phi and theta angles a von Mises-Fisher blur is given by $f(x;\ \mu,\kappa) = N(\kappa)\exp[\kappa\ \mu^T x]$ where $N(\kappa)$ is a normalizing constant, μ is a unit vector on a 3D sphere corresponding to the phi and theta angles from the crystal structure, x is a smoothed unit vector, and κ is the inverse variance chosen to be 100.

Next, we converted those probability distributions to energy landscapes (ie - negative log likelihoods) and sought to minimize the expected energy. This soft restraint encouraged the network to seek out the consensus structure, while still allowing deviations where needed. Specifically, we formulated the fold-specific loss as:

$$L_{fold} = \sum_{x \in \{d,\omega,\theta,\phi\}} \left[\sum_{i,j=1}^{L} \sum_{k=1}^{N_x} - m_{ij}\, p_{x,ijk}\, ln(s_{x,ijk}) \right] / \sum_{i,j=1}^{L} m_{ij}$$

$$m_{ij} = \{\, 1\ if\ i\ and\ j\ are\ \ in\ a\ TERM;\ else\ 0$$

where *p* is the network prediction and *s* is the smoothed probability distribution of the conserved residue pairs. For the second part of the loss function and similar to previous work[19], we sought to maximize the Kullback–Leibler (KL) divergence between the predicted probability distribution and a background distribution for all i,j residue pairs not in a TERM.

$$L_{hall} = - \sum_{x \in \{d,\omega,\theta,\phi\}} \left[\sum_{i,j=1}^{L} \sum_{k=1}^{N_x} (1 - m_{ij})\, p_{x,ijk}\, ln(p_{x,ijk}/b_{x,ijk}) \right] / \sum_{i,j=1}^{L} (1 - m_{ij})$$

where *b* is the background distribution and N_x is the number of bins in each probability distribution (N_d = 37, $N_{\omega,\theta,}$ = 25, N_φ = 13). Briefly, *b* is calculated by a network of similar architecture to trRosetta trained on the same training data, except it is never given sequence information as an input. The final loss is given by:

$$L = L_{fold} + L_{hall}$$

We used a Markov Chain Monte Carlo (MCMC) procedure to search for sequences that trRosetta predicted to fold into structures that minimize this loss function. We allowed four types of moves with different sampling probabilities: mutations (p=0.55), insertions (p=0.15), deletions (p=0.15), and moving segments (p=0.15). Mutations randomly changed one amino acid to another, with an equal transition probability for all 20 amino acids. Insertions inserted a new amino acid (all equally likely) into a random location subject to the KL-divergence loss. Deletions deleted a random residue from the same locations. Finally, we also allowed "segments" to move, cutting and pasting themselves from one part of the sequence to another, while maintaining the same overall segment order. Here, a "segment" is a continuous stretch of amino acids all subject to fold specific loss, often composed of a single strand or helix. Starting from a random sequence of an initial length (typically 120 amino acids), we used the standard Metropolis criteria to accept or reject moves:

$$A_i = min[1, exp(-(L_i - L_{i-1})\ /\ T)]$$

where A_i is the chance of accepting the move at step i, L_i is the loss at the current step, L_{i-1} is the loss at the previous step and T is the temperature. The temperature started at 0.2 and was reduced by half every 5k steps. Generally, it took 30k steps to converge.

3.3 Structure-conditioned multiple sequence alignment

Given the complexity of the NTF2-like protein fold, we hypothesized that it was necessary to impose sequence design rules to disfavor alternative states (negative design). Towards this end, we computed a structure-conditioned multiple sequence alignment based on native NTF2-like proteins. Specifically, we used TMalign[45] to superimpose each of the 2005 predicted native structures (from 3.1) onto each hallucinated backbone (from 3.2). Next, to find structurally corresponding positions, we implemented a structure-based dynamic programming algorithm, similar to the Needleman-Wunsch algorithm[46]. However, instead of using the amino acid similarity as the scoring metric, we used a tunable structure-based score function. After aligning the two structures, we scored the structural similarity of any two residues by empirically weighting several metrics: (1) Distance between Ca atoms, (2) differences between backbone torsion angles (phi and psi) backbone torsion angles, and (3) the angle (degrees) between the vectors pointing from Cα to Cβ in each residue. To calculate the unweighted score for each component, we normalized each by a maximum possible value (180 degrees for angles and 10Å for distances) and included a "set point" that was approximately delineated when we judged a metric to indicate two residues to be more similar than not. Values above this setpoint are positive, indicating two residues are similar and values below the set point indicated two residues are dissimilar.

$$Score_{unweighted} = (set\_point - value) / max\_value$$

Each value was scaled by its normalized weight and summed to give an overall similarity score between any two amino acids.

Score component	Weight	Setpoint	Max value
Phi	1.0	45 degrees	180 degrees
Psi	1.0	45 degrees	180 degrees
CaCb angle	2.0	30 degrees	180 degrees
CaCa distance	2.5	3 Å	10 Å

These similarity scores were used as the similarity metric in our dynamic programming algorithm, in place of the typical BLOSUM62 similarity metric. We used a gap penalty of 0.1 and an extension penalty of 0.0. Finally, after concatenating all the structure-conditioned aligned sequences, we used PSI-BLAST-exB[47,48] to compute sequence redundancy weighted log-odds scores for each amino acid at each position (position-specific scoring matrices, PSSMs).

3.4 Sequence design

To design the resulting backbones, we sought, in addition to the sequence patterns captured in the PSSM (3.3), to further specify the backbone conformation and functionalize the pocket, by installing entire hydrogen bonding networks from native NTF2-like proteins. We compiled two sets of hydrogen bonding networks: a set for the cavity containing 85 networks and another set of networks connecting the C-terminal region of the first helix with the third beta-strand containing 25 networks. In 20 independent attempts for each backbone, we randomly grafted a network from each set, fixed the identities of hydrogen bonding residues, and designed the sequences for all other positions under PSSM constraints. The resulting models were filtered for various backbone

quality metrics and for maintenance of hydrogen bonding networks in the absence of constraints, resulting in a total of 1615 idealized scaffolds.

4. RifDock tuning files

The hierarchical search framework of RifDock is a powerful way to search through 6-dimensional rigid body orientations. While originally designed to work with physics-based forcefields, the scoring machinery can easily be modified to do other things. A system was added called "Tuning Files" that allows one to tune the energetics of RifDock by "requiring" specific interactions. Specified interactions can range from specific hydrogen bonds, to specific bidentates, and even to specific hydrophobic interactions. The specifics are that during the RifGen stage, each stored rotamer is compared against a list of definitions in the Tuning File. If the rotamer satisfies a definition, it is stored into the RIF with a "Requirement Number". Later during RifDock, these Requirement Numbers are available during scoring and the presence or absence of certain rotameric interactions may be used to penalize or even completely discard dock solutions. In this work, the Tuning Files were used to require the specific hydrogen bond interactions between the arginine and the secondary amine in the pyrazine ring of the colenterazine-like substrate. The documentation for RifDock tuning files can be found here:
https://github.com/rifdock/rifdock/blob/master/help/some_rifdock_documentation.md#requireme nttuning_file-information

5. Designing theozyme architectures into *de novo* NTF2 scaffolds

De novo design of luciferases can be divided into three main steps – scaffold construction, substrate placement with required interactions, and sequence design. With the idealized NTF2-like scaffolds in hand, we manually selected 5 diverse anionic DTZ conformers and used the Rotamer Interaction Field (RIF) docking method[29] to exhaustively search a large space of interacting side chains to the anionic form of DTZ. Chemically, deprotonation of N1 hydrogen is the first step to forming an anionic species (**Extended Data Fig. 1**). We first generated RIF using RifGen[29] to sample the placement of amino acid sidechains around DTZ. We required the placement of a positively charged Arginine sidechain by a tuning file (see below) to stabilize the formation of negatively charged N1 atom where the deprotonation initially occurs and enumerated large numbers of possible sidechain interactions with the rest of DTZ. As a comparison, we also placed tryptophan or histidine next to the N1 atom in another tuning file.

```
HBOND_DEFINITION
N1 1 ARG
END_HBOND_DEFINITION

REQUIREMENT_DEFINITION
1 HBOND N1 1
END_REQUIREMENT_DEFINITION
```

RifDock was then used to hierarchically search for the best combination of RIF to place on the input backbone. Although the negative charge can move to another electronegative atom O1 via resonance of the imidazopyrazinone core, it is unclear which anionic species is more critical for the luciferase-catalyzed luminescence emission. Thus, we let RifDock place the polar rotamers on the basis of hydrogen-bond geometry to O1 and apolar rotamers to DTZ without specific requirements. In the next docking step, we parsed the -scaffold_res argument with a list of residue numbers as scaffold backbone positions that were annotated as pocket residues to allow a hierarchical search of RIF placement. We allowed the RIF placements in the pocket residues including or excluding pre-defined hydrogen bond networks. After RifDock, we continued for Rosetta sequence design where the score function was reweighted for higher

buried_unsat_penalty[49], and the amino acid selection was biased by giving a pre-generated PSSM file via SeqprofConsensus task operation. This would minimize buried unsatisfied residues and increase pre-organized architectures in the core that are known to be beneficial for a catalytic pocket[50]. Two rounds of Rosetta FastDesign calculation were included: we restricted the RIF residues and core HBNets to repacking in the first round while we allowed the re-design of other residues based on PSSM during the Monte Carlo simulated annealing procedure. After the surrounding residues were optimized to retain the RIF interactions, we enabled the re-design of RIF residues, giving Rosetta a chance to find efficient aromatic and hydrophobic packing around DTZ while catalytic residues (the N1 requirement) were still limited to only repacking. The final set of designs was obtained after filtering by Rosetta ligand-binding interface energy, shape complementarity, contact molecular surface, number of HbondsToResidue, and the presence of N1 hydrogen bond.

6. Structure prediction of LuxSit with AlphaFold2 and comparison to design model

To computationally assess the accuracy of our LuxSit design model, we performed single sequence structure prediction using AlphaFold2. All models were run with 12 recycles and generated models were relaxed using AMBER[51]. The model with the highest pLDDT was used for comparison to the Rosetta design model and structural superpositions were performed using the Theseus alignment tool to determine backbone RMSD between the design model and AlphaFold2 model[31].

7. Computational design and characterization of de novo luciferases for h-CTZ

To customize a shape complementarity catalytic pocket that can accommodate and catalyze chemiluminescence of another structurally distinct luciferin substrate (2-deoxycoelenterazine, h-CTZ), we sought to use a more diverse set of scaffolds. We first used the deep-learning based protein sequence design method, ProteinMPNN[32] to redesign the whole sequences of the hallucinated NTF2 scaffolds described in 3.2 and the de novo NTF2-like superfamily reported previously[21]. Next, the protein structures of all resulting ProteinMPNN sequences were predicted by AlphaFold2[31]. 6234 scaffolds with diverse pocket geometries were obtained by filtering the pLDDT score greater than 92. With these scaffolds in hand, we selected three different h-CTZ conformers and used the RifDock design strategy described above to search for the sidechain rotamer placements in these scaffolds. Since we've learned from LuxSit design that the N1-Arg and O1-His interactions are critical for catalyzing luminescence emission, both interactions were set as requirements in a tuning file (see an example below) to ensure all RifDock outputs have N1-Arg and O1-His interactions.

```
HBOND_DEFINITION
N1 1 ARG
O1 1 HIS
END_HBOND_DEFINITION

REQUIREMENT_DEFINITION
1 HBOND N1 1
2 HBOND O1 1
END_REQUIREMENT_DEFINITION
```

At this stage, we generated ~215k RifDock outputs in which we subsequently fixed the N1-Arg and O1-His interactions (by applying atom constraints) and allowed Rosetta to redesign all residues within 4 Å of the ligand. The resulting Rosetta designs were prefiltered by contact molecular surface (>350), Rosetta ddG (<-50), and the presence of N1-Arg and O1-His interactions. The prefiltered Rosetta sequences (~30k) were then optimized by ProteinMPNN while all amino acid identities within 4Å of the ligand were kept fixed. All ProteinMPNN sequences

were predicted by AlphaFold2 to obtain predicted 3D protein models where we evaluated the pLDDT score (>85), Cα RMSD (<1.2 Å) to the corresponding Rosetta model, and the numbers of hydrogen bonds to both hypothetical catalytic Arg (>=2) and His (>=1) residues for preorganization. Finally, 46 sequences passed the filters, and we ordered them as eBlocks gene fragments for experimental characterization.

Each synthetic gene was inserted into a modified pET29b vector between two BsaI sites (Golden gate assembly) and transformed into BL21 competent E. coli. The cells were inoculated in LB and grew in a 96-deep well plate with 1mM IPTG at 37 °C for 16h. For the luciferase activity screening, the cells were harvested by centrifugation at 3,800g for 5 min and the pellets were lysed by the BugBuster reagent. Cell lysates were collected by centrifugation at 4000g for 20 min. The His-tag proteins were captured by nickel magnetic beads from cell lysates and bound proteins were eluted in elution buffer (20 mM Tris-HCl pH 8.0, 300 mM NaCl, 300 mM imidazole). The protein concentrations were determined by Bradford assay and the activity of each luciferase was evaluated individually in the presence of 1 μM purified protein and 25 μM h-CTZ in PBS. Through this process, we identified two designs (HTZ3-D2 and HTZ3-G4) that showed luciferase activity and substrate selectivity to h-CTZ. We scaled up the protein expression by the general procedure described above and the purified proteins were used for the characterization shown in **Extended Data Fig. 4**. Serially diluted h-CTZ was mixed with 500 nM HTZ3-D2 or HTZ3-G4 in PBS and the concentration-dependent luminescence was recorded for 30 mins (0.1 s integration and measurements were taken every 1 min). All data points were plotted as the average of the first 10 mins light output and fitted to the Michaelis-Menten equation.

8. Computational SSM experiment to estimate mutation binding free energy

Rosetta cartesian_ddg application[52,53]was used to computationally estimate enzyme and substrate binding free energy. The LuxSit design model was relaxed beforehand in cartesian space with the substrate-bound. For the 21 positions that were experimentally screened for single mutation effects on luciferase activity, each residue was computationally mutated into other amino acid types. Packing and cartesian relaxation were subsequently performed to evaluate the final score in REU. This procedure was applied three times in parallel for both substrate-bound and apo-states. The average of the three calculation results was used to calculate the relative binding free energy (ddG_{bind}) by subtracting the total score of the apo-state from the complex state. The change of binding free energy upon mutation was plotted against experimental activity for every position in **Extended Data Fig. 5**. The wild-type system shown in red dots underwent the same amount of computation by keeping the residue type but applying sidechain repacking and relaxation. The ddG_{bind} rank of the wild type among the mutations is shown with a colored heat map in **Extended Data Fig. 5c**.

9. Construction and screening of designed luciferase libraries

The construction of assembled gene libraries was described previously in detail[54]. In brief, the amino acid sequences of all designed luciferases were first reverse-translated into E. coli codon-optimized DNA sequences. All DNA sequences were categorized into multiple sub-pools by the gene length (~500 designs per sub-pool). Each gene was subsequently split into two fragments (fragment A and fragment B) and added outer and inner primer sequences to the 5' and 3' end (e.g., Outer_oligoA_5primer + design_half_A + Inner_oligoA_3primer and Inner_oligoB_5primer + design_half_B + Outer_oligoB_3primer). All oligos were ordered in one Twist 250nt Oligo Pool. To construct the library of each sub-pool, polymerase chain reaction (PCR) with oligoA_5primer/oligoA_3primer or oligoB_5primer/oligoB_3primer oligonucleotide pairs (see **Table S3**) was used to amplify the individual fragment A or fragment B from each sub-pool. The pool-specific sequences were removed with Uracil Specific Excision Reagent (USER) followed by NEB End Repair kit. Outer primers (oligoA_5primer and oligoB_3primer) were then used for

fragment A and fragment B assembly and amplification. The assembled full-length fragment was digested with XhoI/HindIII and ligated into a predigested pBAD/His B vector. All ligation products were used to transform ElectroMAX™ DH10B Cells, which were next plated on 150 mm × 15 mm LB agar plates supplemented with carbenicillin and L-arabinose. To validate the naïve library, we sequenced 30 random colonies and 11 of the sequences were in our designed library. The plates (~2000 colonies per plate) were incubated at 37 °C overnight to form bacterial colonies and left at 4 °C for another 24 h. To directly image luminescence activity from bacterial colonies, we sprayed the PBS solution containing 30 μM DTZ to each agar plate, waited for 2 min, and the luminescence images were acquired and processed with Bio-Rad ChemiDoc XRS+. After screening 15 plates, active colonies were collected for sequencing, protein expression, and other downstream characterization where LuxSit was selected from three active designs showing catalytic signals above the background.

10. Construction and evaluation of LuxSit site saturation mutagenesis libraries

To create libraries of each single amino acid substitution at residues 13, 14, 17, 18, 35, 37, 38, 49, 52, 53, 56, 60, 65, 81, 83, 94, 96, 98, 100, 110, and 112, forward oligos mixture with degenerate codons (NDT, VHG, and TGG = 1:1:0.1 ratio) and an overlapped reverse oligo were used to amplify the plasmid of LuxSit (**Table S4**). The resulting PCR products were circularized by Gibson Assembly protocol and were subsequently used to transform ElectroMAX™ DH10B Cells. The cells were plated on 150 mm × 15 mm LB agar plates supplemented with carbenicillin and L-arabinose, incubated at 37 °C overnight, and left at 4 °C for another 24 h. As described in the screening of luciferase libraries, colony-based screening by spraying DTZ solution was used to identify active colonies. Inactive colonies were also randomly picked. As a result, a total of 32 colonies were picked for each residue library. 32 × 21 individual colonies were grown in 1 mL of TB supplemented with carbenicillin and L-arabinose in 96-well deep-well culture plates. The plates were shaken at 37 C overnight (~16-18 h) on 96-well plate shakers at 1,100 rpm. Cells were pelleted by centrifugation at 3,800g for 15 min in a tabletop centrifuge. Media was discarded and the cell pellets were resuspended in 0.2 mL BugBuster HT Protein Extraction buffer. The plates were transferred back to 96-well plate shakers and incubated at 1,100 rpm for an additional 30 min. Cellular debris was pelleted again by centrifugation at 4,000g for 15 min, soluble lysates were transferred to a new semi-deep 96-well plate, and incubated with 10 μL of magnetic Ni-NTA beads for 30 min to allow binding. The magnetic extractor was used to first transfer the beads from the binding plates to wash plates with 200 μL IMAC wash buffer in each well, and then transfer the beads to elusion plates containing 30 μL IMAC elution buffer in each well. The concentrations of all proteins in each well were determined by the Bradford assay directly. The elution solution in each well was used to make a 25 μL protein solution at indicated concentration and mixed with 25 μL of 50 μM DTZ PBS solution. The luminescence signals were acquired over a course of 15 min while the actual point mutation was identified by sequencing. Thus, the mutation-to-activity relationship can be mapped. To evaluate whether these beneficial mutations are synergistic, we ordered individual mutants with combinatorial mutations at residue 14, 60, 96, 98, and 110 (see **Table S1**), expressed, and purified these LuxSit variants for kinetic, emission spectra, and luminescence intensity. We identified four mutants that can produce 47 to 77-fold more photons than the parent LuxSit. We assigned one of which, LuxSit-f (A96M/M110V), for its strong initial flash emission. Since the mutations at residue 96 and 110 are robust and mutations at residue 60 are versatile, we generated a fully randomized library at 60, 96, and 110 positions to exhaustively screen all possible combinations. After the colony-based screening, we identified many colonies with strong luciferase activities with DTZ (**Extended Data Fig. 6**). Among all selected mutants, Arg60 is confirmed to be mutable, Ala96 prefers larger hydrophobic sidechains (Leu, Ile, Met, and Cys), and Met110 favors hydrophobic residues (Val, Ile, and Ala). A newly discovered mutant R60S/A96L/M110V with more than 100-fold higher photon flux over LuxSit was assigned LuxSit-i for its high brightness.

11. *In vitro* characterization of photoluminescence properties

For Michaelis–Menten kinetics measurements, 25 μL of serial diluted DTZ substrate in Tris pH 8.0 buffer was added into the wells of a white 96-well half-area microplate containing 25 μL of purified luciferases (final enzyme concentration: 100 nM; substrate concentration: 0.78 to 50 μM). Measurements were taken every 1 min (0.1 s integration and 10 s shaking between each interval) for a total of 20 min. Initial velocities were estimated as the average of the light intensities from the first three data points to fit the Michaelis-Menten equation. All relative arbitrary unit (RLU) per second values were converted to photon/s by the luminol-H_2O_2-HRP calibration method[42]. Following the equation: I_{max} = LQY × k_{cat} × [E], I_{max} is the maximal photon flux (photon s^{-1}), [E] is the total enzyme concentration, and V_{max} is the maximum photon flux per molecule (photon s^{-1} $molecule^{-1}$) from the fitting of the Michaelis-Menten equation. To determine the luminescent quantum yields, 125 pmol of individual substrate in 25 μL PBS was injected into 25 μL PBS containing 100 nM corresponding luciferase. DTZ was used for all LuxSit variants while CTZ was used as the substrate of native RLuc. The luminescence signals were monitored until the reactions were completed (0.1 s integration and measurements were taken every 5 s for a total of 40 min). The sum of luminescence photon counts was normalized to the total photon counts of RLuc/CTZ pair (LQY = 5.3 ± 0.1%)[55] to derive relative luminescent quantum yields of LuxSit variants (**Extended Data Fig. 7c**). k_{cat} values for each individual enzyme were calculated using the equation: k_{cat} = V_{max} / LQY. To record emission spectra, 25 μL of 50 μM DTZ in PBS was injected into 25 μL of 200 nM pure luciferases and the emission spectra were collected with 0.1 s integration and 2 nm increments from 300 to 700 nm. *In vitro* luminescence activity measurements of LuxSit-i expressing HEK293T or HeLa cells were done similarly as 15,000 intact cells or lysates were used in the assay instead of purified luciferases. To evaluate the substrate specificity, 25 μL of 50 μM substrate analogs in PBS was added to 25 μL of 200 nM indicated luciferases, and the signals were recorded over 20 min. Data were shown as the total luminescence signal over the first 10 min. We normalized the data by setting the highest emission substrate at 100%. The unnormalized plot of **Fig. 4c** was attached as **Fig. S4.**

12. Circular dichroism (CD)

Purified protein samples were prepared at 15 μM in pH 7.4 10 mM phosphate buffer. Spectra from 190 nm to 260 nm were recorded at 25 °C, 50 °C, 75 °C, 95 °C, and after cooling back to 25 °C. Thermal denaturation was monitored at 220 nm from 25 °C to 95 °C (1°C per min increments). Tm values were not reported because no obvious inflection points of the melting curves.

13. Mammalian cell culture and transfection

HEK293T and HeLa cell lines were maintained at 37 °C with humidified 5% CO_2 atmosphere and cultured in Dulbecco's Modified Eagle's Medium (DMEM, Gibco) supplemented with 10% fetal bovine serum (FBS, Sigma). Cells were transfected with Turbofectin 8.0 (Origene) with 500 μg of plasmid DNA. After 24 h at 37 °C in a CO_2 incubator, the medium was removed, and cells were collected and resuspended in Dulbecco's phosphate-buffered saline (DPBS).

14. Fluorescence Microscopy and image analysis

Cells were washed twice with HBSS and subsequently imaged in HBSS in the dark at 37°C. Right before imaging, cells were incubated with 25 μM DTZ. Epifluorescence imaging was conducted on a Yokogawa CSU-X1 microscope equipped with a Hamamatsu ORCA-Fusion scientific CMOS camera and Lumencor Celesta light engine. Objectives used were: 10×, NA 0.45, WD 4.0 mm, 20×, NA 1.4, WD 0.13 mm, and 40×, NA 0.95, WD 0.17–0.25 mm with correction collar for cover glass thickness (0.11 mm to 0.23 mm) (Plan Apochromat Lambda). Imaging for BFP utilized a 408 nm laser, 432/36 nm dichroic, and a 440/40 nm emission filter (Semrock). Exposure times

were 200 ms for BFP and 10 s for luminescence. All epifluorescence experiments were subsequently analyzed using NIS Elements 5.30 software.

15. Multiplex dual-luciferase reporter assay for the cAMP/PKA and NF-κB pathways

HEK293T cells were grown in a tissue culture-grade white 96-well plate and transfected with indicated CRE-RLuc, NFκB-LuxSit-i, and CMV-CyOFP plasmids. 24 h after transfection, the medium was replaced by 2 μM of Forskolin (FSK) or 300 ng/mL human tumor necrosis factor alpha (TNFα) in regular cell media. 23 h after stimulation, the cells were resuspended in DPBS by pipette mixing. 25 μL of DPBS containing 30,000 intact cells was mixed with 25 μL of CelLytic M for 15 min to make cell lysates. For intact cell assay, 25 μL of DPBS containing 15,000 intact cells was mixed with 25 μL of PP-CTZ (2μM) or/and DTZ (10μM) in DPBS. For cell lysate assay, 25 μL of cell lysate was added to 25 μL of PP-CTZ (2μM) or/and DTZ (10μM) to initiate luminescence reactions. The signals were recorded every 1 min for a total of 10 min. The light signals were collected in the substrate-resolved mode without filters and with 528/20 and 390/35 filters under the spectrally resolved mode. Area scanning of the CyOFP fluorescence intensity at 480 nm (excitation wavelength) and 580 nm (emission wavelength) was used to estimate the total cell numbers and transfection efficiency. The reported unit was the average of the first 10 min luminescence (RLU) over the relative fluorescence units (a.u.). To derive fold-of-activation, all values were normalized to the corresponding non-stimulated control.

16. Quantum mechanics calculation for the energy profile of DTZ luminescence

All calculations were carried out by using the Gaussian 16 Rev. A 03. Given the benchmark study done by Martin Head-Gorden and co-workers recently[56], geometry optimizations and frequency calculations were performed at the (u)ωB97X-D/6-31+G(d) level of theory[57,58]. Frequency calculations were conducted at the same level of theory to confirm the presence of local minima (no imaginary frequencies) and transition states (one imaginary frequency) on the PES. Subsequent single-point energies were computed at the (u)ωB97X-D/def2TZVP level. Solvent effect was modeled by employing the CPCM model. Conformational searches were conducted using the CREST conformer-rotamer ensemble sampling tool version 2.10.2 with xtb version 6.3.3 to ensure the substrate showed are lowest energy conformers[59,60]. Intrinsic reaction coordinate (IRC) calculations were performed to verify that the saddle points found were true TSs connecting the reactants and the products. TDDFT result were given by using the same level and method with DFT part, setting nstates=10 to ensure the calculation accuracy. All thermodynamic quantities (1 mol/L, 298.15 K) were computed in the GoodVibes code[61] with quasiharmonic corrections[62]. 3D renderings of stationary points were generated using open-source PyMOL.

17. Molecular dynamics simulations of proposed enzyme-ligand complexes

Molecular dynamics simulations were performed using the GPU code (pmemd)[63] of the AMBER 16 software package. Substrate structures were optimized at the B3LYP-D3/6-31G(d) level of theory[64–66] using Gausian16, Revision A.03. Rosetta parameter files for the substrates were generated using the provided molfile2params.py script on the optimized structures. Substrate starting positions were determined using the ligand_dock application[67–69] of the Rosetta3 software package. Each substrate was docked using 500 trajectories starting from the original design position. Docked structures with the lowest interface_delta and ligand_auto_rms_no_super were chosen for simulation starting coordinates. AMBER parameter files for the substrates were generated using the general AMBER force field (GAFF)[70]. Partial charges were set to fit the electrostatic potential generated at the previously mentioned level of theory using the RESP model[71]. Charges were calculated using the Merz-Singh-Kollman scheme[72,73] using Gausian16, Revision A.03. The system was immersed in a pre-equilibrated octahedral box with a 10 Å buffer of TIP3P water molecules[74] using the tleap module. Explicit Na^+ and Cl^- ions were added to neutralize the total charge of the system. All subsequent calculations were performed using the

AMBER 14 force field (ff14sb)[75]. Two minimization steps were conducted in serial, each consisting of 2,500 steepest descent steps and 2,500 conjugate gradient steps. Protein and substrate atoms were restrained with a force constant of 500.0 kcal mol^{-1} $Å^{-2}$ in the first minimization step, allowing the solvent to minimize. Sidechain atoms were unrestrained in the second minimization, but backbone and substrate atoms were restrained with a force constant of 50.0 kcal mol^{-1} $Å^{-2}$. Minimizations were followed by two equilibration steps where backbone and substrate atoms were restrained with a force constant of 30.0 kcal mol^{-1} $Å^{-2}$. In the first equilibration step, the system was heated from 0-300 K over 300 ps using constant-volume and periodic-boundary conditions and a 1fs time step. The SHAKE algorithm was used to constrain bonds involving hydrogens. Long-range electrostatic effects were modeled using the particle-mesh-Ewald method[76] and an 8 Å cutoff was applied to the Lennard-Jones and electrostatic interactions. A second equilibration was then conducted for 50 ns using a 2-fs time step at constant pressure using isotropic position scaling with the Monte Carlo barostat and a pressure relaxation time of 5.0 ps. Production trajectories were conducted the same as equilibration 2, however, all atoms were unrestrained during production. Production trajectories were propagated in pentaplicate from the ending of the second equilibrium and each simulation ran for 500 ns. Simulations were analyzed using the cpptraj module[77] to extract relevant information, including PDB files for visualization. Plots were generated using custom python scripts invoking matplotlib and seaborn. PDB files were visualized, and figures were created, using open-source PyMOL.

<u>Statistical analysis and reproducibility</u>

No statistical methods were used to pre-determine the sample size. No sample was excluded from the data analysis. Results were reproduced using different batches of pure proteins on different days. Coomassie-stained SDS PAGE gels were done at least twice for each experiment. Microscopic fluorescence and luminescence images were repeated in biological triplicate with similar results. Unless otherwise indicated, data are shown as mean ± s.d., and error bars in figures represent s.d. of technical triplicate. Data were analyzed and plotted using GraphPad Prism 8, seaborn, and matplotlib.

Supplementary tables and figures:

Fig. S1. Gel source data. This gel presents the recombinant expression of LuxSit in E. coli.

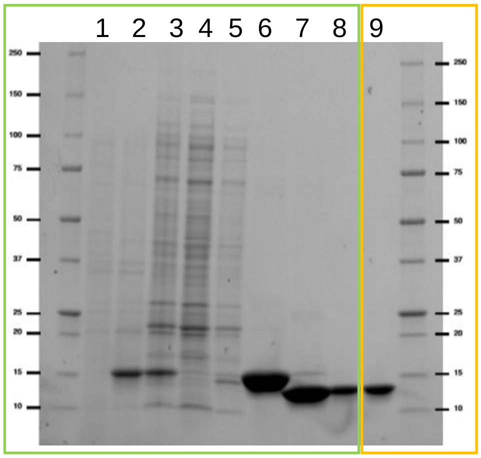

Annotations for each lane – 1: Pre-IPTG; 2: Post-IPTG; 3: Soluble lysate; 4: Flow-through; 5: Wash; 6: Elusion; 7: Post-TEV cleavage; 8: Post-SEC; 9: Post-SEC and concentrated.
The left part (green) of the gel was cropped as Extended Data Fig. 3a.
The right part (yellow) of the gel was cropped as Fig. 2a.

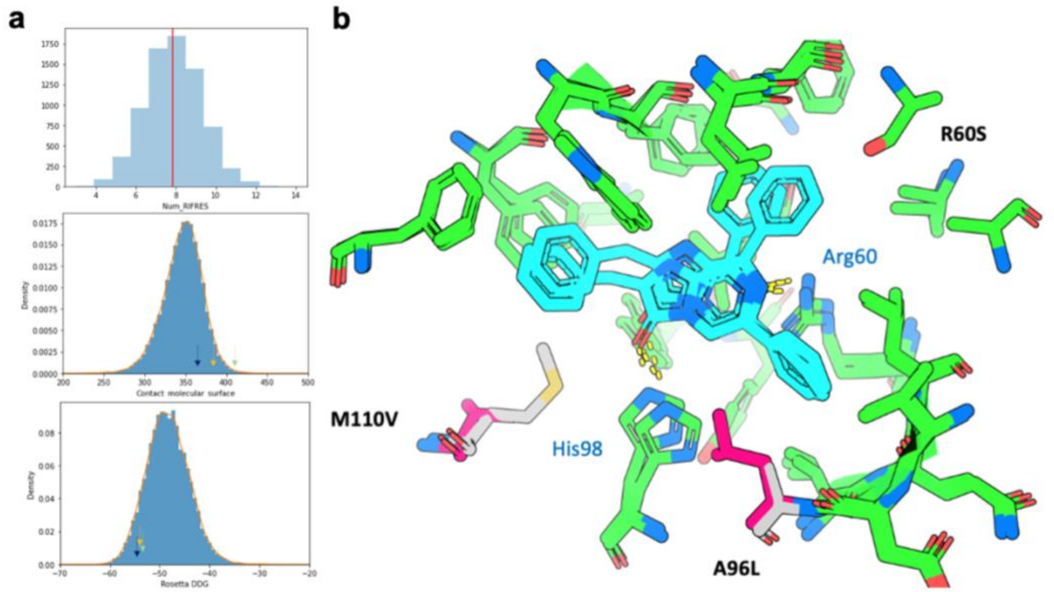

Fig. S2. Additional computational analysis of designed luciferases. a, Histogram plots of 7648 luciferase designs show the number of sidechains placed on each scaffold by RifDock (top, the red line indicates the average) and *in silico* scores, such as contact molecular surface (middle) and Rosetta ddG (bottom). Three active DTZ designs we experimentally identified (blue, green, and yellow arrows indicate 15078, 21093, and LuxSit designs, see **Extended Data Fig. 2**) were located on the upper end of both distributions, suggesting that having substantial packing and contact with the ligand is an important factor for active luciferases. **b**, Superimposed protein sidechain and DTZ interactions of LuxSit and Luxsit-I (Full atom RMSD = 1.2 Å). Mutations (R60S, A96L, and M110V) of LuxSit-i were colored in magenta. For the ligand interactions of LuxSit, ten sidechains were initially placed from RifDock, and five of them were mutated during RosettaDesign. Both Tyr-His and Asp-Arg dyads were from the pre-defined hydrogen bond networks.

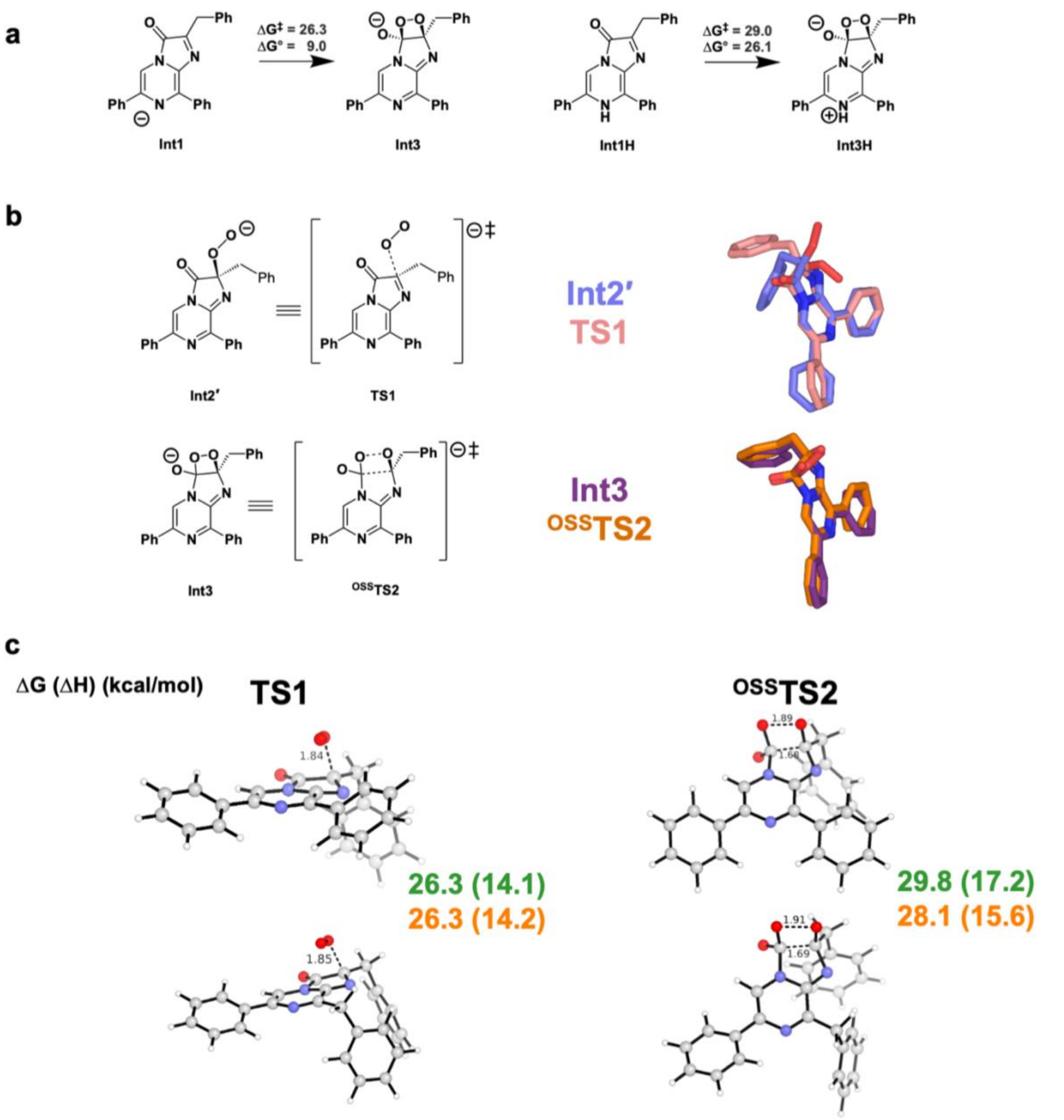

Fig. S3. Additional QM and MM computational models used in this study. a, Comparison of energies for the first proposed step indicates that deprotonation of N1 is critical for the reaction to proceed. **b**, Overlay of Int2′ and TS1 (top) and overlay of Int3 and ^{OSS}TS2 (bottom) highlighting the structural similarities between transition states and their analogs. While Int2′ does not lie on the intrinsic reaction path connecting TS1 and Int3, its structural similarity to TS1 makes it a useful transition state analog for ground state MD simulations. **c**, Comparison of TS1 (left) and ^{OSS}TS2 (right) for DTZ (top) and bis-CTZ (bottom) pathways. Computed transition state energies are nearly identical, suggesting that the observed selectivity is entirely due to the protein.

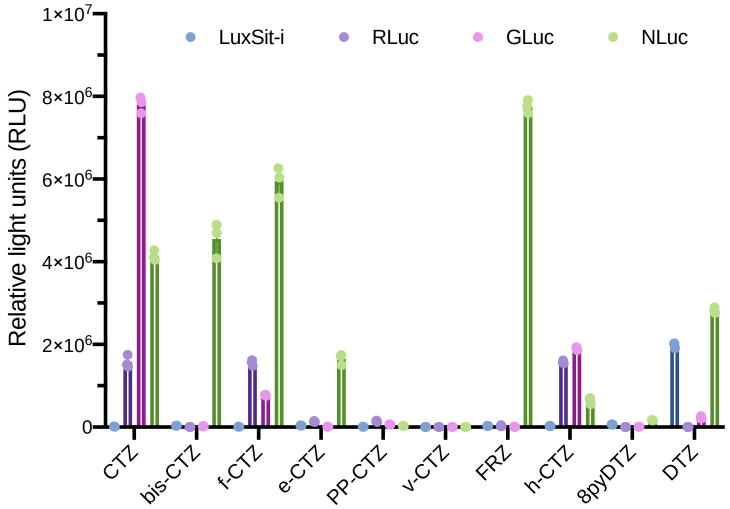

Fig. S4. The unnormalized light outputs plot of Fig. 4c. Indicated luciferin analogs (25 μM) were mixed with 100 nM of either LuxSit-i, RLuc, GLuc, or NLuc, and the total luminescence signal over the first 10 min was plotted (n=3). We normalized the data by setting the highest emission substrate at 100% in each group to create **Fig. 4c**.

Table S1. Enzymatic and photoluminescence properties of LuxSit and its mutants

	λ_{max} (nm)	K_M (µM)[a]	V_{max} (x10^{-2} photon s^{-1} $molecule^{-1}$)[a]	Relative emission intensity[b]
LuxSit (60R/96A/98H/110M)	478	18.3(14.1-21)	0.32(0.29-0.37)	1
Y14E	478	12.6(9.9-16.1)	0.19(0.17-0.21)	0.95
R60C	476	45.2(28.7-78.9)	0.57(0.44-0.8)	1.55
A96I	476	17.5(12.1-25.9)	1.3(1.1-1.5)	4.32
A96M	476	4.8(3.1-7.3)	6.0(5.3-6.9)	16.4
H98N	476	29.3(22.1-39.8)	0.17(0.15-0.2)	0.48
M110C	478	67.5(43-121)	0.4(0.3-0.6)	0.64
M110V	480	30.3(22.7-41.5)	7.1(6.2-8.4)	19.3
60C/96I/98H/110C	476	11.3(6.5-20.2)	0.12(0.1-0.16)	0.39
60R/96I/98N/110C	476	8.7(3-7.4)	0.02(0.017-0.022)	0.12
60R/96I/98H/110C	480	4.3(2,7-6.8)	0.05(0.045-0.059)	0.27
60C/96I/98H/110V	478	6.3(4.8-8.5)	14.7(13.4-16.2)	77.1
60C/96M/98N/110V	474	9.7(5.6-17.4)	0.13(0.1-0.16)	0.5
60C/96M/98H/110C	478	30.9(18.3-57.2)	2.44(1.9-3.4)	4.26
60R/96I/98N/110V	476	7.2(5.4-9.5)	0.32(0.29-0.35)	1.39
60R/96I/98H/110V	482	10.8(8-14.6)	12.9(11.7-14.5)	47.5
60C/96M/98H/110V	476	7.0(4.8-10.2)	15.9(14-18.1)	66.3
14E/60C/96M/98H/110 V	478	8.0(4.0-15.8)	0.16(0.12-0.2)	1.0
60R/96M/98N/110V	478	8.0(5.3-12.2)	0.044(0.04-0.05)	0.21
60C/96M/98N/110C	476	17.4(13.4-22.8)	0.016(0.015-0.018)	0.08
60C/96I/98N/110C	476	24.7(18.7-33.4)	0.03(0.026-0.035)	0.11
60R/96M/98H/110C	474	23.4(15.9-35.8)	0.28(0.24-0.35)	0.58
60C/96I/98N/110V	480	80.9(43.3-222)	0.83(0.55-1.8)	1.81
60R/96M/98H/110V (LuxSit-f)	480	8.9(6.7-11.9)	19.7(17.9-21.9)	60.9
60R/96M/98N/110C	476	9.3(7-12.2)	0.019(0.017-0.021)	0.96
60S/96L/98H/110V (LuxSit-i)	482	2.5(1.9-3.3)	36.1(33.7-38.6)	153

a. mean (95% CI)., n=3; **b**. Integrated luminescence intensity over the first 20 min. All values were normalized to the signal of LuxSit with 25 µM DTZ in Tris pH 8.0 buffer.

Table S2. Photoluminescence properties of selected LuxSit variants

	LQY[a] (%)	k_{cat} (1/s)	K_M (μM)	V_{max} (photon s^{-1} $molecule^{-1}$)	k_{cat} / K_M ($x10^6$ $M^{-1}s^{-1}$)
LuxSit-i	14.5±1.8	2.5	2.5	0.36	1.0
LuxSit-f	10.9±1.5	1.8	8.9	0.20	0.2
RLuc	5.3[b]	4.9	1.5	0.26	3.3

a. mean±s.d., n=3. LQY (luminescent quantum yield) measurements were performed by consuming 125 pmol of DTZ (w/ LuxSit-i, and LuxSit-f) or CTZ (w/ RLuc) in 50 µL PBS with 50 nM corresponding recombinant luciferases. **b**. All LQY values were estimated relative to the reported quantum yield of RLuc[78]. All values were calculated by the assumptions of the simplest Michaelis-Menten kinetics model, excluding potential substrate/product inhibition or enzyme modification during the reaction[79,80].

Table S3. Oligonucleotide sequences for the construction of designed libraries

name	oligoA_5primer	oligoA_3primer	oligoB_5primer	oligoB_3primer
pBAD_TJ_1	AAGGATCCGAGCTCGAGC	AAtcgtttcgcaggcATGG	GCGAgacttgtggccctTT	TAAGCTTGGCTGTTTTGG
pBAD_TJ_2	AAGGATCCGAGCTCGAGC	AAcgggtatgcatgcATGG	GCGActcagctgctcacTT	TAAGCTTGGCTGTTTTGG
pBAD_TJ_3	AAGGATCCGAGCTCGAGC	AAcgggcatcagaggATGG	GCGActtgatgtggcagTT	TAAGCTTGGCTGTTTTGG
pBAD_TJ_4	AAGGATCCGAGCTCGAGC	AAgttgcttcacggcATGG	GCGAaggctctgcttggTT	TAAGCTTGGCTGTTTTGG
pBAD_TJ_5	AAGGATCCGAGCTCGAGC	AAatcccgcgtatgcATGG	GCGAgtcgatggctcccTT	TAAGCTTGGCTGTTTTGG
pBAD_TJ_6	AAGGATCCGAGCTCGAGC	AAggcgtgctaaacgATGG	GCGAgagctattcccggTT	TAAGCTTGGCTGTTTTGG
pBAD_TJ_7	AAGGATCCGAGCTCGAGC	AAccgcccactctacATGG	GCGAaggtttcggcttcTT	TAAGCTTGGCTGTTTTGG
pBAD_TJ_8	AAGGATCCGAGCTCGAGC	AAcagggctgagcgaATGG	GCGAcgagtcgtgggtaTT	TAAGCTTGGCTGTTTTGG
pBAD_TJ_9	AAGGATCCGAGCTCGAGC	AAacccactcgccacATGG	GCGAtgttgcagactggTT	TAAGCTTGGCTGTTTTGG
pBAD_TJ_10	AAGGATCCGAGCTCGAGC	AAcgagggtcgacgtATGG	GCGAggtgtcgcaacatTT	TAAGCTTGGCTGTTTTGG
pBAD_TJ_11	AAGGATCCGAGCTCGAGC	AAcctggcaatccggATGG	GCGAcgagagtctcccaTT	TAAGCTTGGCTGTTTTGG
pBAD_TJ_12	AAGGATCCGAGCTCGAGC	AAggtccggctgatcATGG	GCGAgtggtgtagtggcTT	TAAGCTTGGCTGTTTTGG
pBAD_TJ_13	AAGGATCCGAGCTCGAGC	AAtggggctcaggcaATGG	GCGAtcgcgtgagtggtTT	TAAGCTTGGCTGTTTTGG
pBAD_TJ_14	AAGGATCCGAGCTCGAGC	AAcagtggcatccccATGG	GCGAatatccgccgttgTT	TAAGCTTGGCTGTTTTGG
pBAD_jason_1	AAGGATCCGAGCTCGAGC	AAgcttacgggcaacATGG	GCGAcccactggcataaTT	TAAGCTTGGCTGTTTTGG
pBAD_jason_2	AAGGATCCGAGCTCGAGC	AAcccagtaagggtcATGG	GCGAgcgaccttagagtTT	TAAGCTTGGCTGTTTTGG
pBAD_jason_3	AAGGATCCGAGCTCGAGC	AAgctcccgtatcagATGG	GCGAgagggtctacagaTT	TAAGCTTGGCTGTTTTGG
pBAD_jason_4	AAGGATCCGAGCTCGAGC	AAgctgtcgtgctcaATGG	GCGAgtggcatggcaacTT	TAAGCTTGGCTGTTTTGG
pBAD_jason_5	AAGGATCCGAGCTCGAGC	AAtgcccgagttccaATGG	GCGAccgcgtggttagaTT	TAAGCTTGGCTGTTTTGG
pBAD_jason_6	AAGGATCCGAGCTCGAGC	AAcgctggttgtccaATGG	GCGAgccagttttaggcTT	TAAGCTTGGCTGTTTTGG
pBAD_jason_7	AAGGATCCGAGCTCGAGC	AAgctttgtgggaccATGG	GCGAcggggaattaaccTT	TAAGCTTGGCTGTTTTGG
pBAD_jason_8	AAGGATCCGAGCTCGAGC	AAcacctcacgcaacATGG	GCGAggcgactatagctTT	TAAGCTTGGCTGTTTTGG
pBAD_jason_9	AAGGATCCGAGCTCGAGC	AAccggatacacaggATGG	GCGAgggaacgtaatggTT	TAAGCTTGGCTGTTTTGG
pBAD_jason_10	AAGGATCCGAGCTCGAGC	AAcaagtccttccggATGG	GCGAcgggcattaatccTT	TAAGCTTGGCTGTTTTGG
pBAD_jason_11	AAGGATCCGAGCTCGAGC	AAatgccgtaagccgATGG	GCGAtccactagctggtTT	TAAGCTTGGCTGTTTTGG
pBAD_jason_12	AAGGATCCGAGCTCGAGC	AAcaagcaggtgaccATGG	GCGAgactagcgagtagTT	TAAGCTTGGCTGTTTTGG

Table S4. Oligonucleotide pairs for the construction of SSM libraries

1	
013FB_f	TTCGTCAGTTTCTGCGTCGTndtTATGAAGCGCTGGATAGCGGC
013FZ_f	TTCGTCAGTTTCTGCGTCGTvhgTATGAAGCGCTGGATAGCGGC
013FW_f	TTCGTCAGTTTCTGCGTCGTtggTATGAAGCGCTGGATAGCGGC
013_r	ACGACGCAGAAACTGACGAATCTGTTCTTCGCTCATGCTGC
2	
014YB_f	GTCAGTTTCTGCGTCGTTTTndtGAAGCGCTGGATAGCGGC
014YZ_f	GTCAGTTTCTGCGTCGTTTTvhgGAAGCGCTGGATAGCGGC
014YW_f	GTCAGTTTCTGCGTCGTTTTtggGAAGCGCTGGATAGCGGC
014_r	AAAACGACGCAGAAACTGACGAATCTGTTCTTCGCTCATGCTGC
3	
017LB_f	TGCGTCGTTTTTATGAAGCGndtGATAGCGGCGATGCGGATAC
017LZ_f	TGCGTCGTTTTTATGAAGCGvhgGATAGCGGCGATGCGGATAC
017LW_f	TGCGTCGTTTTTATGAAGCGtggGATAGCGGCGATGCGGATAC
017_r	CGCTTCATAAAAACGACGCAGAAACTGACGAATCTGTTCTTCGCT
4	
018DB_f	GTCGTTTTTATGAAGCGCTGndtAGCGGCGATGCGGATACC
018DZ_f	GTCGTTTTTATGAAGCGCTGvhgAGCGGCGATGCGGATACC
018DW_f	GTCGTTTTTATGAAGCGCTGtggAGCGGCGATGCGGATACC
018_r	CAGCGCTTCATAAAAACGACGCAGAAACTGACGAATCTGTTCTTCG
5	
035IB_f	TGTTTCATCCGGGCGTGACAndtCATCTGTGGGATGGCGTTACC
035IZ_f	TGTTTCATCCGGGCGTGACAvhgCATCTGTGGGATGGCGTTACC
035IW_f	TGTTTCATCCGGGCGTGACAtggCATCTGTGGGATGGCGTTACC
035_r	TGTCACGCCCGGATGAAACAGGCTCGCAGCGGTATCCG
6	
037LB_f	ATCCGGGCGTGACAATTCATndtTGGGATGGCGTTACCTTTACCA
037LZ_f	ATCCGGGCGTGACAATTCATvhgTGGGATGGCGTTACCTTTACCA
037LW_f	ATCCGGGCGTGACAATTCATtggTGGGATGGCGTTACCTTTACCA
037_r	ATGAATTGTCACGCCCGGATGAAACAGGCTCGCAGCGG
7	
038WB_f	CGGGCGTGACAATTCATCTGndtGATGGCGTTACCTTTACCAGCC
038WZ_f	CGGGCGTGACAATTCATCTGvhgGATGGCGTTACCTTTACCAGCC
038WW_f	CGGGCGTGACAATTCATCTGtggGATGGCGTTACCTTTACCAGCC
038_r	CAGATGAATTGTCACGCCCGGATGAAACAGGCTCGCAGCG
8	
049FB_f	CCTTTACCAGCCGTGAAGAAndtCGTGAATGGTTTGAACGTCTGTTTAG
049FZ_f	CCTTTACCAGCCGTGAAGAAvhgCGTGAATGGTTTGAACGTCTGTTTAG
049FW_f	CCTTTACCAGCCGTGAAGAAtggCGTGAATGGTTTGAACGTCTGTTTAG
049_r	TTCTTCACGGCTGGTAAAGGTAACGCCATCCCACAGATGAATTG
9	
052WB_f	GCCGTGAAGAATTTCGTGAAndtTTTGAACGTCTGTTTAGCACCCG
052WZ_f	GCCGTGAAGAATTTCGTGAAvhgTTTGAACGTCTGTTTAGCACCCG
052WW_f	GCCGTGAAGAATTTCGTGAAtggTTTGAACGTCTGTTTAGCACCCG
052_r	TTCACGAAATTCTTCACGGCTGGTAAAGGTAACGCCATCCCA
10	
053FB_f	GTGAAGAATTTCGTGAATGGndtGAACGTCTGTTTAGCACCCGT
053FZ_f	GTGAAGAATTTCGTGAATGGvhgGAACGTCTGTTTAGCACCCGT
053FW_f	GTGAAGAATTTCGTGAATGGtggGAACGTCTGTTTAGCACCCGT
053_r	CCATTCACGAAATTCTTCACGGCTGGTAAAGGTAACGCCATC
11	
056LB_f	TTCGTGAATGGTTTGAACGTndtTTTAGCACCCGTAAAGATGCGC
056LZ_f	TTCGTGAATGGTTTGAACGTvhgTTTAGCACCCGTAAAGATGCGC
056LW_f	TTCGTGAATGGTTTGAACGTtggTTTAGCACCCGTAAAGATGCGC
056_r	ACGTTCAAACCATTCACGAAATTCTTCACGGCTGGTAAAGGTAAC

12	
060RB_f	TTGAACGTCTGTTTAGCACCndtAAAGATGCGCAGCGTGAAATTAAG
060RZ_f	TTGAACGTCTGTTTAGCACCvhgAAAGATGCGCAGCGTGAAATTAAG
060RW_f	TTGAACGTCTGTTTAGCACCtggAAAGATGCGCAGCGTGAAATTAAG
060_r	GGTGCTAAACAGACGTTCAAACCATTCACGAAATTCTTCACGGC
13	
065RB_f	GCACCCGTAAAGATGCGCAGndtGAAATTAAGAGCCTGGAAGTACGTGG
065RZ_f	GCACCCGTAAAGATGCGCAGvhgGAAATTAAGAGCCTGGAAGTACGTGG
065RW_f	GCACCCGTAAAGATGCGCAGtggGAAATTAAGAGCCTGGAAGTACGTGG
065_r	CTGCGCATCTTTACGGGTGCTAAACAGACGTTCAAACCATTCACGA
14	
081VB_f	GCGATACCGTGGAAGTGCATndtCAGTTGCACGCGACCCAT
081VZ_f	GCGATACCGTGGAAGTGCATvhgCAGTTGCACGCGACCCAT
081VW_f	GCGATACCGTGGAAGTGCATtggCAGTTGCACGCGACCCAT
081_r	ATGCACTTCCACGGTATCGCCACGTACTTCCAGGCTCTTAATTTCA
15	
083LB_f	CCGTGGAAGTGCATGTGCAGndtCACGCGACCCATAATGGCC
083LZ_f	CCGTGGAAGTGCATGTGCAGvhgCACGCGACCCATAATGGCC
083LW_f	CCGTGGAAGTGCATGTGCAGtggCACGCGACCCATAATGGCC
083_r	CTGCACATGCACTTCCACGGTATCGCCACGTACTTCCAGGC
16	
094VB_f	ATAATGGCCAGAAACATACCndtGATGCAACCCATCATTGGCATTTTC
094VZ_f	ATAATGGCCAGAAACATACCvhgGATGCAACCCATCATTGGCATTTTC
094VW_f	ATAATGGCCAGAAACATACCtggGATGCAACCCATCATTGGCATTTTC
094_r	GGTATGTTTCTGGCCATTATGGGTCGCGTGCAACTGCA
17	
096AB_f	GCCAGAAACATACCGTAGATndtACCCATCATTGGCATTTTCGTGG
096AZ_f	GCCAGAAACATACCGTAGATvhgACCCATCATTGGCATTTTCGTGG
096AW_f	GCCAGAAACATACCGTAGATtggACCCATCATTGGCATTTTCGTGG
096_r	ATCTACGGTATGTTTCTGGCCATTATGGGTCGCGTGCAACT
18	
098HB_f	AACATACCGTAGATGCAACCndtCATTGGCATTTTCGTGGCAATCG
098HZ_f	AACATACCGTAGATGCAACCvhgCATTGGCATTTTCGTGGCAATCG
098HW_f	AACATACCGTAGATGCAACCtggCATTGGCATTTTCGTGGCAATCG
098_r	GGTTGCATCTACGGTATGTTTCTGGCCATTATGGGTCGCG
29	
100WB_f	CCGTAGATGCAACCCATCATndtCATTTTCGTGGCAATCGTGTGAC
100WZ_f	CCGTAGATGCAACCCATCATvhgCATTTTCGTGGCAATCGTGTGAC
100WW_f	CCGTAGATGCAACCCATCATtggCATTTTCGTGGCAATCGTGTGAC
100_r	ATGATGGGTTGCATCTACGGTATGTTTCTGGCCATTATGGGTCG
20	
110MB_f	GTGGCAATCGTGTGACCGAAndtCGTGTGCATATCAATCCGACCG
110MZ_f	GTGGCAATCGTGTGACCGAAvhgCGTGTGCATATCAATCCGACCG
110MW_f	GTGGCAATCGTGTGACCGAAtggCGTGTGCATATCAATCCGACCG
110_r	TTCGGTCACACGATTGCCACGAAAATGCCAATGATGGGTTGCAT
21	
112VB_f	ATCGTGTGACCGAAATGCGTndtCATATCAATCCGACCGGCTAAGC
112VZ_f	ATCGTGTGACCGAAATGCGTvhgCATATCAATCCGACCGGCTAAGC
112VW_f	ATCGTGTGACCGAAATGCGTtggCATATCAATCCGACCGGCTAAGC
112_r	ACGCATTTCGGTCACACGATTGCCACGAAAATGCCAATGATGG

Table S5. Amino acid and nucleotide sequences of the *de novo* designed and native/engineered luciferases used in this study

The sequences (underlined) below contain a PolyHis-TEV or PolyHis tag for protein purification

>LuxSit
MGSHHHHHHGSGSENLYFQGSMSEEQIRQFLRRFYEALDSGDADTAASLFHPGVTIHLWDGVTFTSREEF
REWFERLFSTRKDAQREIKSLEVRGDTVEVHVQLHATHNGQKHTVDATHHWHFRGNRVTEMRVHINPTG

>LuxSit-f
MGSHHHHHHGSGSENLYFQGMSEEQIRQFLRRFYEALDSGDADTAASLFHPGVTIHLWDGVTFTSREEFR
EWFERLFSTRKDAQREIKSLEVRGDTVEVHVQLHATHNGQKHTVDMTHHWHFRGNRVTEVRVHINPTG

>LuxSit-i
MGSHHHHHHGSGSENLYFQGMSEEQIRQFLRRFYEALDSGDADTAASLFHPGVTIHLWDGVTFTSREEFR
EWFERLFSTSKDAQREIKSLEVRGDTVEVHVQLHATHNGQKHTVDLTHHWHFRGNRVTEVRVHINPTG

>LuxSit-i (codon optimized for E. coli expression)
ATGGGTAGCCATCACCACCATCACCATGGTAGCGGTAGCGAGAACTTGTACTTCCAAGGAATGAGCGAAG
AACAGATTCGTCAGTTTCTGCGTCGTTTTTATGAAGCGCTGGATAGCGGCGATGCGGATACCGCTGCGAG
CCTGTTTCATCCGGGCGTGACAATTCATCTGTGGGATGGCGTTACCTTTACCAGCCGTGAAGAATTTCGT
GAATGGTTTGAACGTCTGTTTAGCACCAGTAAAGATGCGCAGCGTGAAATTAAGAGCCTGGAAGTACGTG
GCGATACCGTGGAAGTGCATGTGCAGTTGCACGCGACCCATAATGGCCAGAAACATACCGTAGATTTGAC
CCATCATTGGCATTTTCGTGGCAATCGTGTGACCGAAGTTCGTGTGCATATCAATCCGACCGGC

>LuxSit-i (codon optimized for human cell expression)
ATGAGCGAGGAGCAGATCAGACAGTTCCTGAGGAGATTCTACGAGGCCCTGGATAGCGGAGACGCCGACA
CAGCTGCCAGCCTTTTCCATCCTGGCGTGACCATCCACCTGTGGGACGGCGTCACCTTCACTAGCAGGGA
GGAGTTCAGGGAGTGGTTCGAGAGACTGTTCAGCACCAGCAAGGACGCCCAGAGAGAGATCAAGAGCCTG
GAAGTTAGAGGCGACACCGTGGAAGTGCACGTGCAGCTGCACGCCACACACAACGGACAGAAGCACACCG
TCGACCTGACCCACCACTGGCACTTCAGAGGCAACAGAGTGACCGAGGTGAGAGTGCACATCAATCCCAC
CGGC

>HTZ3-D2
MSGSPKEKIKKIAWEIIESINEKDIEKFLSYFTDDVVFTNHKNETITSKEELREQREKYLSKNATYHVEE
ITVGEDNVATIKGYVTVDNITKPSIVYLKMNDEDKVIEAEVVLIGSHHHHHH

>HTZ3-G4
MSGSPEDERLKELVETAAKAALKGDAEALKKIHHPDFVAKDGDKEWKGVDATAKVIQDYYDKHPDVKVTA
YVEEVKAEGDIGVAKMKVVFTVGDEVVEEDALVTGEKIDGTWYLTEFERRGSHHHHHH

>*Renilla* luciferase (RLuc)
MGSHHHHHHGSGSENLYFQGSGMTSKVYDPEQRKRMITGPQWWARCKQMNVLDSFINYYDSEKHAENAVI
FLHGNAASSYLWRHVVPHIEPVARCIIPDLIGMGKSGKSGNGSYRLLDHYKYLTAWFELLNLPKKIIFVG
HDWGACLAFHYSYEHQDKIKAIVHAESVVDVIESWDEWPDIEEDIALIKSEEGEKMVLENNFFVETMLPS
KIMRKLEPEEFAAYLEPFKEKGEVRRPTLSWPREIPLVKGGKPDVVQIVRNYNAYLRASDDLPKMFIESD
PGFFSNAIVEGAKKFPNTEFVKVKGLHFSQEDAPDEMGKYIKSFVERVLKNEQ

>*Gaussia* luciferase (GLuc)
MGSHHHHHHGSGSENLYFQGSGKPTENNEDFNIVAVASNFATTDLDADRGKLPGKKLPLEVLKEMEANAR
KAGCTRGCLICLSHIKCTPKMKKFIPGRCHTYEGDKESAQGGIGEAIVDIPEIPGFKDLEPMEQFIAQVD
LCVDCTTGCLKGLANVQCSDLLKKWLPQRCATFASKIQGQVDKIKGAGGD

>NanoKaz (or NLuc) engineered from *Oplophorus* luciferase
MGCSHHHHHHGSGSENLYFQGSVFTLEDFVGDWRQTAGYNLDQVLEQGGVSSLFQNLGVSVTPIQRIVLS
GENGLKIDIHVIIPYEGLSGDQMGQIEKIFKVVYPVDDHHFKVILHYGTLVIDGVTPNMIDYFGRPYEGI
AVFDGKKITVTGTLWNGNKIIDERLINPDGSLLFRVTINGVTGWRLCERILA

Supplementary Notes:

Design scripts for family-wide hallucination are available here:
https://files.ipd.uw.edu/pub/luxSit/scaffold_generation.tar.gz
Find detailed instructions in a README file.

Hallucinated NTF2 scaffolds are available here:
https://files.ipd.uw.edu/pub/luxSit/scaffolds.tar.gz
There are 1615 pdb and pssm files for each pdb.

We described below the computational methods toward the active de novo luciferase, LuxSit:

RifGen and RifDock software are available here: https://github.com/rifdock/rifdock
The main/source folder and the compiled Rifdock is referred to as $ROSETTA and $RIFDOCK.
RifGen/RifDock methods are mainly modified from Cao et. al, Nature 2022, 605, 551-560
Example files for this section can be downloaded from here:
https://files.ipd.uw.edu/pub/luxSit/luciferase_designs_methods.zip
Four steps were included: (1) RifGen, (2) RifDock, (3) RosettaDesign, and (4) Filtering.

Step 1. RifGen

To run RifGen: $RIFDOCK/build/apps/rosetta/rifgen @rifgen.flags > rifgen.log 2>&1

rifgen.flags file contains the following:
[target.pdb] – The target substrate pdb file
[target.params] – The Rosetta params file for the target substrate
[require_hb.txt] – A tuning file that specifies the requirements
[output_dir] – output directory
[output.rif.gz] – output file name

```
# DTN is the 3-letter name of anionic DTZ
# In this example: [target.pdb]=DTN_10.pdb; [target.params]=DTN_rotlib.params; [require_hb.txt]=
require_hb.txt

################################## File I/O flags ##################################

-rifgen:target          [target.pdb]
-tuning_file            [require_hb.txt]
-extra_res_fa           [target.params]
-rifgen:outdir          [output_dir]
-rifgen:outfile         [output.rif.gz]

################################## RIF Flags ##################################

-rifgen::rif_type RotScoreSat

############################## Normal RIF Configuration #########################

-rifgen:apores ALA VAL ILE LEU PHE TYR TRP
-rifgen:donres SER THR TYR TRP ASN GLN HIS LYS ARG
-rifgen:accres SER THR TYR  HIS DTN
-rifgen:score_threshold -0.5
```

```
################################## General flags ##################################

-rifgen:hbond_weight 2.0
-min_hb_quality_for_satisfaction -0.25

# Change this depending on the database of the Rosetta you compiled for RifDock
-database [database path]

#################################################################################
#################### END OF USER ADJUSTABLE SETTINGS #####################
#################################################################################

-rifgen:extra_rotamers true
-rifgen:extra_rif_rotamers true
-rif_accum_scratch_size_M 24000
-renumber_pdb
-hash_cart_resl     0.7
-hash_angle_resl    14.0
-rifgen::rosetta_field_resl 0.25
-rifgen::search_resolutions 3.0 1.5 0.75
# A cache directory. Populated on the first run and then never changes.
-rifgen:data_cache_dir    ./cache_dir
-rifgen:score_cut_adjust 0.8
-hbond_cart_sample_hack_range 0.33
-hbond_cart_sample_hack_resl  0.33
-rifgen:tip_tol_deg        60.0
-rifgen:rot_samp_resl       6.0
-rifgen:rif_hbond_dump_fraction  0.00001
-rifgen:rif_apo_dump_fraction    0.000001
-add_orbitals
-rifgen:beam_size_M 10000.0
-rifgen:hash_preallocate_mult 0.125
-rifgen:max_rf_bounding_ratio 4.0
-rifgen:hash_cart_resls   16.0   8.0   4.0   2.0   1.0
-rifgen:hash_cart_bounds   512   512   512   512   512
-rifgen:lever_bounds      16.0   8.0   4.0   2.0   1.0
-rifgen:hash_ang_resls     38.8  24.4  17.2  13.6  11.8
-rifgen:lever_radii        23.6 18.785501 13.324600  8.425850  4.855575

#################################################################################
```

Step 2. Rifdock

To run Rifdock: $RIFDOCK/build/apps/rosetta/rif_dock_test @rifdock.flags -scaffolds [input_scaffold.pdb] -scaffold_res [input_scaffold.pos] > rifdock_logs.log 2>&1

In this example: the [input_scaffold.pdb]=inp_1502.pdb,which is a renamed scaffold of 6983_1_0.25_5_14_2978_2_0.35_1_Y14_Y82_D101_S115d2gexa1_clean_0001_N18.rd1.pdb

[input_scaffold.pos] = inp_1502.pos, which listed what residues were allowed to dock
13 17 25 29 35 37 53 54 57 61 64 80 84 86 95 97 99 113 117 119

rifdock.flags file contains the following:

```
########################### what you need for docking ###########################
# Fill this in with the output from the log file of Rifgen
-rif_dock:target_pdb
-in:file:extra_res_fa
-rif_dock:target_rf_resl
-rif_dock:target_rf_cache
-rif_dock:target_bounding_xmaps
-rif_dock:target_bounding_xmaps
-rif_dock:target_bounding_xmaps
-rif_dock:target_bounding_xmaps
-rif_dock:target_bounding_xmaps
-rif_dock:target_rif
-rif_dock:target_donors
-rif_dock:target_acceptors
-rif_dock:extra_rotamers
-rif_dock:extra_rif_rotamers
-rif_dock:rot_spec_fname

############################### Constant paths ################################

# Change the -database and -rifdock:rotrf_cache_dir to paths appropriate on your system.

-database /home/me/rifdock/rifdock_rosetta/main/database

# A cache directory. Populated on the first-run and then never changes.
-rif_dock:rotrf_cache_dir /home/me/rifdock/cache

############################ Flags that control output ############################

-rif_dock:outdir  ./
-rif_dock:dokfile all.dok
-rif_dock:n_pdb_out 30
-rif_dock:redundancy_filter_mag 1.0
-rif_dock:align_output_to_scaffold true
-output_full_scaffold

##################### Flags that affect runtime/search space ########################

-beam_size_M 5
-hsearch_scale_factor 1.2
-rif_dock:global_score_cut 0.0


################### Flags that affect how things are scored #########################

-hbond_weight 2.0
-upweight_multi_hbond 0.0
-min_hb_quality_for_satisfaction -0.1
-scaff_bb_hbond_weight 2.0
-favorable_1body_multiplier 0.2
-favorable_1body_multiplier_cutoff 4
-favorable_2body_multiplier 5
-user_rotamer_bonus_constant 0
-user_rotamer_bonus_per_chi 0
-rif_dock:upweight_iface 2.0
```

```
################ stuff related to picking designable and fixed positions ##################

-scaffold_res_use_best_guess false
-rif_dock::dont_use_scaffold_loops false
-rif_dock:scaffold_to_ala false
-rif_dock:scaffold_to_ala_selonly true
-rif_dock:replace_all_with_ala_1bre false

############################### HackPack options #################################
-hack_pack true
-rif_dock:hack_pack_frac  0.1

########################### rosetta re-scoring / min stuff ############################

-rif_dock:rosetta_score_cut -5.0
-rif_dock:rosetta_score_fraction 0.006
-rif_dock:rosetta_min_fraction 0.07
-rif_dock:replace_orig_scaffold_res false
-rif_dock:rosetta_min_scaffoldbb false
-rif_dock:rosetta_min_targetbb   false
-rif_dock:rosetta_hard_min false
-rif_dock:rosetta_min_at_least 30
-rif_dock:rosetta_score_rifres_rifres_weight   0.6
-rif_dock:rosetta_score_rifres_scaffold_weight 0.4

######################## Special flags that do special things #########################

-require_satisfaction    1
-require_n_rifres  2
-requirements 1

####################### options to favor existing scaffold residues #####################

-add_native_scaffold_rots_when_packing 0 # 1
-bonus_to_native_scaffold_res          0 # -0.5

############################## Twobody table caching #############################

-rif_dock:cache_scaffold_data false
-rif_dock:data_cache_dir  ./rifdock_scaffdata

################################################################################
##################### END OF USER ADJUSTABLE SETTINGS ######################
################################################################################

-beta
-score:weights beta_soft
-add_orbitals false
-rif_dock:pack_n_iters    2
-rif_dock:pack_iter_mult  2.0
-rif_dock:packing_use_rif_rotamers        true
-rif_dock:always_available_rotamers_level 0
-rif_dock:rotrf_resl   0.25
-rif_dock:rotrf_spread 0.0
-rif_dock:rotrf_scale_atr 1.0
-rif_dock::rf_resl 0.5
```

-rif_dock::rf_oversample 2
-rif_dock:use_scaffold_bounding_grids 0
-rif_dock:target_rf_oversample 2
-mute core.scoring.ScoreFunctionFactory
-mute core.io.pose_from_sfr.PoseFromSFRBuilder

##

Step 3. RosettaDesign

To run RosettaDesign, you need the following variables and input files:
[pocket_res] – a list of pocket residues
[HBNET_res] – a list of pre-defined hydrogen bond networks
[rifres_res] – a list of RIF residues
[lig_res] – the ligand residue number
[cat_res] – the hypothetical catalytic residue numbers
[pssm_file] – a standard Rosetta position-specific scoring matrix (PSSM) file for that scaffold

One example to run this protocol: $ROSETTA/bin/rosetta_scripts -beta_nov16 -preserve_header true -parser:protocol design_w_pssm_rifdock.xml -extra_res_fa [target.params] -s [input.pdb] -holes:dalphaball $ROSETTA/external/DAlpahBall/DAlphaBall.gcc -parser:script_vars pocket_res="13,17,25,29,35,37,53,54,57,61,64,80,84,86,95,97,99,113,117,119" HBNET_res="14,18,66,82,101,115" lig_res="123" cat_res="35" rifres_res="13,17,29,35,37,53,54,57,86,95,97,113,117,119" pssm_file=[pssm_file]

In this example: [input.pdb]=inp_1502_000000029.pdb; [target.params]=DTN_rotlib.params; [pssm_file]=inp_1502.pssm; and design_w_pssm_rifdock.xml is the RosettaScript xml

Step 4. Filtering

```
# Use the following script to parse all .sc files
# wd=[working_path_to_folders]
# Assuming each folder contains designs for each scaffold

# This script combines all score files in all_scores.txt

from glob import glob
counter=0
dirs=glob(wd)
write_header=1
with open("all_scores.txt","w") as out:
    for d in dirs:
        sc_files=glob(f"{d}/design_*score.sc")
        if len(sc_files) == 1:
            counter += 1
            sc_file=sc_files[0]
            all_lines=[]
            with open(sc_file, 'r') as inp:
                for line in inp:
                    if line.startswith("SCORE:"):
                        if line.strip().split()[1] != "total_score":
                            line=line.strip().split()
                            line=line[:]+[d]
                            line="\t".join(line)
```

```
                all_lines.append(line)
            else:
                line=line.strip().split()
                line=line[:]+["directory"]
                line="\t".join(line)
                all_lines.append(line)
    if write_header:
        out.write("\n".join(all_lines[0:])+"\n")
        write_header=0
    else:
        out.write("\n".join(all_lines[1:])+"\n")

print("Processed directories: {counter}".format(counter=counter))

# The following script sets up the filtering and prints the design pdb files

import pandas as pd
df = pd.read_csv(all_scores.txt,delim_whitespace=True,header=0)
best=df[(df.contact_molecular_surface > 350) &
    (df.filt_sc >= 0.75) &
    (df.hbonds2lig >= 2) &
    (df.N1_hbond_fil >= 1) &
    (df.ddg_norepack <= -40)]

pdbs=[]
for i,j in best.iterrows():
  pdbs.append("{f}.pdb".format(f=j.description))
print(pdbs)
```

Design models of LuxSit and LuxSit-i are available online in LuxSit_models.zip:
luxsit.pdb – the originally designed model of LuxSit
luxsiti_relaxed.pdb – LuxSit-i model built by Rosetta

Atomic coordinates of the structures used in the DFT calculations:

1

-1 1
C -1.246644 -1.594445 -0.567477
C -2.280262 -0.781826 -0.211328
N -2.122882 0.579101 -0.054370
C -0.941878 1.131896 -0.260803
C 0.183991 0.357002 -0.640116
N 1.457040 0.655089 -0.876585
C 2.066919 -0.520477 -1.188375
C 1.185188 -1.631482 -1.140727
O 1.325856 -2.871140 -1.326815
N -0.021815 -1.024792 -0.792547
C 3.533766 -0.612930 -1.486262
C 4.378398 -0.878566 -0.252662
C 4.185323 -2.053761 0.484072
C 4.941342 -2.309804 1.623110
C 5.903595 -1.392854 2.049017
C 6.100459 -0.220534 1.325358
C 5.341532 0.032288 0.182020
H 5.500781 0.951001 -0.377982
H 6.845119 0.501370 1.649192
H 6.493487 -1.592554 2.938940
H 4.780820 -3.227321 2.182805
H 3.426593 -2.758749 0.151339
H 3.869119 0.313021 -1.966342
H 3.692856 -1.427344 -2.204631
C -0.845936 2.604759 -0.057317
C 0.143600 3.381629 -0.672305
C 0.184212 4.759387 -0.473300
C -0.757273 5.382431 0.341829
C -1.745849 4.616543 0.958364
C -1.790566 3.241975 0.757583
H -2.560298 2.641400 1.230539
H -2.483280 5.091824 1.598819
H -0.721491 6.456797 0.497074
H 0.955864 5.348133 -0.961016
H 0.885331 2.898366 -1.296908
C -3.637491 -1.322901 0.053612
C -4.507006 -0.638525 0.911663
C -5.776457 -1.136928 1.186744
C -6.204863 -2.329412 0.606829
C -5.352607 -3.013932 -0.257069
C -4.084719 -2.512643 -0.535688
H -3.447066 -3.044192 -1.236363
H -5.679677 -3.936574 -0.727730
H -7.196162 -2.718090 0.820114
H -6.433221 -0.592274 1.859204
H -4.172497 0.290398 1.361721
H -1.293646 -2.671126 -0.672308

2

-1 3
C -1.198752 -1.621041 -0.544589
C -2.234362 -0.802332 -0.210112
N -2.072450 0.559212 -0.054816
C -0.886706 1.103336 -0.247013
C 0.241670 0.320035 -0.602661
N 1.520616 0.616171 -0.818495
C 2.129082 -0.556532 -1.122996
C 1.242379 -1.670323 -1.078283
O 1.387335 -2.907816 -1.249327

N 0.033743 -1.058924 -0.744130
C 3.597022 -0.655862 -1.409283
C 4.428561 -0.901521 -0.163015
C 4.236780 -2.070408 0.583704
C 4.979452 -2.306621 1.735769
C 5.926837 -1.375682 2.164311
C 6.122344 -0.209425 1.430449
C 5.376637 0.023673 0.274433
H 5.534354 0.937752 -0.293416
H 6.855657 0.523081 1.756252
H 6.506439 -1.559814 3.064271
H 4.820026 -3.219447 2.303304
H 3.489673 -2.786736 0.249158
H 3.936365 0.261891 -1.901659
H 3.758443 -1.482399 -2.112663
C -0.783117 2.576889 -0.064595
C 0.174635 3.343318 -0.739321
C 0.222900 4.723895 -0.564138
C -0.679114 5.358231 0.286412
C -1.636643 4.602147 0.961329
C -1.689729 3.224503 0.783763
H -2.435903 2.630649 1.301424
H -2.342974 5.087434 1.628667
H -0.637198 6.434857 0.423279
H 0.968290 5.306064 -1.098269
H 0.882685 2.850083 -1.394391
C -3.599457 -1.334843 0.029459
C -4.481245 -0.643579 0.869177
C -5.758328 -1.134634 1.121139
C -6.181994 -2.326071 0.535671
C -5.317352 -3.017049 -0.310590
C -4.041684 -2.523303 -0.566030
H -3.393790 -3.059835 -1.253370
H -5.640493 -3.938846 -0.785547
H -7.179228 -2.708964 0.730925
H -6.424816 -0.584973 1.779779
H -4.150608 0.284691 1.323329
H -1.249998 -2.697568 -0.648572
O 0.549982 -0.374891 -3.734206
O -0.199959 0.538438 -3.456816

TS1
-1 1
C -0.793428 -1.493122 -0.373176
C -1.821085 -0.683578 -0.010039
N -1.631736 0.682890 0.163252
C -0.475672 1.231075 -0.061664
C 0.666119 0.443270 -0.497134
N 1.952039 0.754506 -0.564888
C 2.494257 -0.343053 -1.159296
C 1.626838 -1.536119 -0.981961
O 1.801825 -2.737403 -1.151949
N 0.436692 -0.934145 -0.564463
C 3.963743 -0.466054 -1.421918
C 4.749139 -0.719485 -0.152198
C 4.740616 -1.984482 0.444727
C 5.435575 -2.215598 1.628315
C 6.150537 -1.183356 2.234378
C 6.162373 0.080207 1.648952
C 5.464524 0.308564 0.464313
H 5.475800 1.297368 0.012486
H 6.716234 0.890947 2.113650

H	6.694793	-1.363637	3.156759
H	5.420428	-3.204184	2.078437
H	4.172339	-2.784627	-0.022633
H	4.303412	0.455455	-1.903731
H	4.114475	-1.288063	-2.129904
C	-0.340285	2.697476	0.095498
C	0.469293	3.433025	-0.777384
C	0.557070	4.815569	-0.645528
C	-0.151003	5.471896	0.359444
C	-0.960985	4.742051	1.228722
C	-1.060131	3.361715	1.093733
H	-1.691290	2.785444	1.763283
H	-1.514727	5.249474	2.012993
H	-0.074158	6.550109	0.464475
H	1.178793	5.383242	-1.331586
H	1.002264	2.907376	-1.562297
C	-3.190568	-1.194277	0.232584
C	-4.059400	-0.489711	1.074680
C	-5.342058	-0.963941	1.329787
C	-5.782764	-2.149841	0.746055
C	-4.930229	-2.852986	-0.102453
C	-3.649149	-2.376813	-0.362333
H	-3.012097	-2.922147	-1.052840
H	-5.267183	-3.770177	-0.576398
H	-6.784543	-2.519005	0.943914
H	-5.999352	-0.404873	1.989466
H	-3.717060	0.434681	1.527915
H	-0.848949	-2.568180	-0.492312
O	1.691797	-0.276226	-2.809036
O	0.644392	0.547987	-2.723222

3

-1 1

C	-0.692973	-1.488501	-0.897862
C	-1.621249	-0.744577	-0.213042
N	-1.347523	0.562596	0.078042
C	-0.227287	1.141137	-0.268214
C	0.794176	0.390765	-1.000906
N	1.998818	0.755158	-1.343481
C	2.605392	-0.371690	-2.005004
C	1.642171	-1.617217	-1.959143
O	1.835322	-2.788971	-1.556661
N	0.434663	-0.899067	-1.310745
C	4.073509	-0.560694	-1.663970
C	4.351709	-0.499940	-0.179501
C	3.776441	-1.434448	0.689847
C	4.020849	-1.372297	2.058489
C	4.847381	-0.378687	2.582551
C	5.424899	0.554020	1.725416
C	5.175195	0.492243	0.355191
H	5.625750	1.227806	-0.307028
H	6.069525	1.333567	2.121729
H	5.038345	-0.332192	3.650823
H	3.564463	-2.103157	2.720621
H	3.122670	-2.199364	0.275986
H	4.647591	0.214360	-2.184907
H	4.385397	-1.529046	-2.072353
C	-0.033532	2.558107	0.135172
C	0.946013	3.377971	-0.440821
C	1.071113	4.709483	-0.050071
C	0.230264	5.243345	0.921299
C	-0.745765	4.433858	1.503072

C	-0.876931	3.108055	1.112984
H	-1.637274	2.479066	1.563044
H	-1.405387	4.837737	2.265599
H	0.333924	6.280414	1.226582
H	1.833929	5.330852	-0.510358
H	1.613904	2.961904	-1.184454
C	-2.910166	-1.296983	0.260959
C	-3.576663	-0.696303	1.336614
C	-4.780404	-1.211295	1.805538
C	-5.343458	-2.338530	1.210248
C	-4.693052	-2.940084	0.135136
C	-3.492258	-2.421206	-0.339473
H	-3.018117	-2.888106	-1.198146
H	-5.127330	-3.811213	-0.346593
H	-6.282887	-2.741053	1.576889
H	-5.278807	-0.731912	2.643220
H	-3.137701	0.179130	1.803989
H	-0.779217	-2.544757	-1.129487
O	2.419304	-0.313308	-3.439322
O	1.476583	-1.434985	-3.439673

$^{OSS}TS_{2}$

-1 1

C	-0.743517	-1.502981	-0.758279
C	-1.679100	-0.728214	-0.123280
N	-1.393560	0.582700	0.147835
C	-0.269341	1.141466	-0.210530
C	0.741249	0.372468	-0.944395
N	1.913622	0.746525	-1.356075
C	2.511513	-0.368023	-2.058751
C	1.616327	-1.740812	-1.700360
O	1.947081	-2.681906	-0.940676
N	0.400082	-0.940008	-1.176864
C	4.016410	-0.521822	-1.813801
C	4.423222	-0.509747	-0.360577
C	4.662326	-1.700740	0.329467
C	5.035745	-1.687699	1.671980
C	5.176925	-0.477914	2.347299
C	4.942919	0.717668	1.669322
C	4.571167	0.698810	0.328139
H	4.383442	1.632091	-0.194962
H	5.052924	1.667136	2.185900
H	5.469582	-0.465517	3.393318
H	5.217296	-2.625200	2.190281
H	4.538173	-2.645378	-0.190539
H	4.514703	0.298867	-2.344334
H	4.328257	-1.455874	-2.291781
C	-0.051584	2.557501	0.186369
C	0.924744	3.368124	-0.407855
C	1.073793	4.696657	-0.015182
C	0.260203	5.236764	0.975725
C	-0.713136	4.436888	1.574936
C	-0.867813	3.114060	1.183380
H	-1.625304	2.492206	1.648005
H	-1.351567	4.845575	2.352767
H	0.382864	6.271305	1.282576
H	1.833850	5.310712	-0.489609
H	1.569513	2.947183	-1.168860
C	-2.989266	-1.250667	0.324741
C	-3.667203	-0.630377	1.381978
C	-4.892030	-1.117581	1.825171
C	-5.465012	-2.235702	1.221949

C -4.803439 -2.855982 0.164517
C -3.581522 -2.364520 -0.284835
H -3.098669 -2.842911 -1.132332
H -5.245552 -3.719401 -0.323923
H -6.421121 -2.616210 1.568408
H -5.399508 -0.623711 2.648873
H -3.220428 0.238119 1.854971
H -0.847228 -2.565789 -0.946873
O 2.165548 -0.433470 -3.359592
O 1.399727 -2.134309 -3.031020

4
-1 1
C -1.621756 -1.849065 -0.646394
C -2.467111 -0.970265 -0.038579
N -1.976823 0.220148 0.433052
C -0.749542 0.568320 0.182596
C 0.142749 -0.251830 -0.655107
N 1.264315 0.239495 -1.067371
C 2.042652 -0.245296 -2.081214
C 0.589390 -2.716807 -1.332995
O 1.744411 -2.624702 -0.919758
N -0.322539 -1.530680 -0.904114
C 3.547093 -0.247589 -1.811036
C 3.966728 0.261158 -0.456434
C 3.786301 -0.529828 0.683243
C 4.137097 -0.051278 1.941892
C 4.675936 1.227933 2.082693
C 4.861913 2.021290 0.953662
C 4.510672 1.537350 -0.305638
H 4.655756 2.164055 -1.182573
H 5.282228 3.018255 1.051943
H 4.948035 1.602658 3.065208
H 3.985930 -0.675496 2.818262
H 3.334513 -1.511075 0.569922
H 4.022257 0.329871 -2.612088
H 3.857989 -1.288805 -1.946347
C -0.282120 1.860782 0.754956
C 1.019467 2.043508 1.238930
C 1.382130 3.245316 1.839149
C 0.460970 4.284402 1.954129
C -0.834326 4.112106 1.470354
C -1.203363 2.907428 0.881420
H -2.213744 2.766219 0.511072
H -1.560047 4.916034 1.551191
H 0.750669 5.223534 2.416427
H 2.394135 3.364850 2.214968
H 1.752555 1.250948 1.146229
C -3.892782 -1.273055 0.219532
C -4.560190 -0.649091 1.280741
C -5.897027 -0.929889 1.541975
C -6.593339 -1.839616 0.748526
C -5.940910 -2.460147 -0.314595
C -4.605313 -2.174664 -0.581391
H -4.122606 -2.642456 -1.434995
H -6.477092 -3.159257 -0.949570
H -7.637518 -2.057226 0.951744
H -6.395714 -0.439208 2.372880
H -4.017498 0.056507 1.901043
H -1.895122 -2.854867 -0.936702
O 1.621667 -0.549094 -3.193355
O -0.019782 -3.574004 -1.969216

TS$_3$

-1 1

C	-1.675115	-1.867084	-0.690085
C	-2.524601	-0.977022	-0.062905
N	-2.017644	0.178441	0.424594
C	-0.754861	0.489197	0.222191
C	0.116305	-0.369105	-0.563005
N	1.362216	0.006433	-0.826872
C	2.060142	-0.421071	-1.895433
C	0.699085	-3.141291	-1.415998
O	1.692213	-3.059702	-0.763851
N	-0.391837	-1.575475	-0.923166
C	3.584044	-0.265203	-1.765840
C	4.068027	0.325284	-0.468886
C	4.127369	-0.459791	0.686878
C	4.523967	0.086998	1.903038
C	4.877911	1.434044	1.983122
C	4.832594	2.223193	0.836787
C	4.430909	1.669778	-0.377844
H	4.391025	2.294747	-1.267106
H	5.108571	3.272844	0.887097
H	5.189661	1.862766	2.931193
H	4.558688	-0.537924	2.791188
H	3.836769	-1.505727	0.629047
H	3.928260	0.329257	-2.620054
H	3.995939	-1.272052	-1.905522
C	-0.288864	1.772043	0.823425
C	1.020270	1.966903	1.286215
C	1.378858	3.163264	1.901396
C	0.449243	4.188833	2.055150
C	-0.853248	4.006812	1.593841
C	-1.217698	2.808631	0.989791
H	-2.234153	2.662604	0.638919
H	-1.587485	4.799877	1.703179
H	0.737079	5.123845	2.527259
H	2.398431	3.289439	2.254411
H	1.761041	1.188801	1.147894
C	-3.962727	-1.252972	0.159233
C	-4.644323	-0.626794	1.210521
C	-5.993053	-0.880810	1.434509
C	-6.688085	-1.766574	0.612757
C	-6.022041	-2.389537	-0.440170
C	-4.673734	-2.131176	-0.668826
H	-4.180009	-2.602685	-1.513829
H	-6.555834	-3.071361	-1.095682
H	-7.741064	-1.965324	0.788210
H	-6.502927	-0.388002	2.257333
H	-4.102187	0.060567	1.851677
H	-1.993586	-2.855885	-1.007554
O	1.612224	-0.852707	-2.966685
O	0.007334	-3.742269	-2.178630

5

-1 1

C	-0.861297	-1.525589	0.043247
C	-1.926050	-0.644281	0.203121
N	-1.696670	0.677383	0.110472
C	-0.472110	1.109536	-0.154493
C	0.627278	0.195283	-0.327153
N	1.886110	0.676211	-0.466332
C	2.805643	0.053458	-1.217284

N 0.382743 -1.129694 -0.212925
C 4.239158 0.542360 -0.935603
C 4.887579 -0.357950 0.089696
C 5.662164 -1.453276 -0.301932
C 6.217516 -2.311562 0.644935
C 6.002525 -2.088336 2.003086
C 5.226911 -1.001952 2.405609
C 4.673933 -0.147305 1.455947
H 4.062070 0.693928 1.771261
H 5.053156 -0.819380 3.462451
H 6.437127 -2.754380 2.742770
H 6.820317 -3.155277 0.320594
H 5.831069 -1.633356 -1.360689
H 4.211338 1.571845 -0.567736
H 4.806706 0.514850 -1.870937
C -0.315419 2.589124 -0.232599
C 0.531693 3.209531 -1.159219
C 0.608112 4.597589 -1.231615
C -0.150778 5.391701 -0.374340
C -0.997297 4.785384 0.551677
C -1.081711 3.398237 0.615852
H -1.747651 2.925174 1.331035
H -1.593143 5.393638 1.226279
H -0.083257 6.474451 -0.427663
H 1.264885 5.060659 -1.962662
H 1.133506 2.598083 -1.820354
C -3.308778 -1.088423 0.502165
C -4.193227 -0.226749 1.163985
C -5.490691 -0.628311 1.462200
C -5.932243 -1.901433 1.105065
C -5.064632 -2.764663 0.440114
C -3.767246 -2.361055 0.137268
H -3.116379 -3.039062 -0.407157
H -5.400702 -3.754434 0.144968
H -6.945004 -2.215868 1.338779
H -6.159050 0.053986 1.979696
H -3.848218 0.763604 1.443534
H -1.006669 -2.599096 0.155302
O 2.642924 -0.813409 -2.093467

TS_3*
-1 1
C -1.576489 -1.810273 -0.649267
C -2.465556 -0.895093 -0.048638
N -2.061299 0.303853 0.392872
C -0.722722 0.621990 0.215504
C 0.107875 -0.219405 -0.611364
N 1.256257 0.242368 -1.072429
C 2.008682 -0.312123 -2.069227
C 0.856356 -3.092119 -1.375595
O 1.898941 -2.867132 -0.851479
N -0.309100 -1.486672 -0.936547
C 3.519078 -0.305480 -1.830229
C 3.965881 0.239844 -0.498290
C 3.835711 -0.527896 0.663290
C 4.204172 -0.010890 1.901759
C 4.714595 1.282835 1.997975
C 4.856007 2.052519 0.845935
C 4.482704 1.532255 -0.391756
H 4.588339 2.141491 -1.286250
H 5.255419 3.060734 0.909601
H 5.001775 1.687313 2.964078

H 4.092007 -0.618770 2.795074
H 3.424156 -1.530404 0.590203
H 3.970231 0.258372 -2.654461
H 3.838116 -1.346102 -1.951771
C -0.272749 1.845496 0.838407
C 1.089304 2.186898 1.058595
C 1.437386 3.366929 1.695263
C 0.460826 4.255482 2.156542
C -0.884594 3.928380 1.975038
C -1.248101 2.751276 1.339554
H -2.294386 2.506645 1.198700
H -1.659340 4.602231 2.332773
H 0.743738 5.175914 2.659138
H 2.490996 3.586319 1.848918
H 1.870521 1.508868 0.742107
C -3.883875 -1.247369 0.147956
C -4.654164 -0.509850 1.061805
C -5.993413 -0.814583 1.274014
C -6.595370 -1.859054 0.574575
C -5.845411 -2.593441 -0.343854
C -4.506379 -2.290172 -0.558336
H -3.953239 -2.855733 -1.301209
H -6.308509 -3.400805 -0.903222
H -7.642019 -2.096894 0.739229
H -6.570130 -0.236092 1.989928
H -4.177289 0.297960 1.605542
H -1.878475 -2.814554 -0.925962
O 1.561178 -0.681970 -3.150782
O 0.115455 -3.786036 -1.990794

1H
0 1
C -1.235982 -1.608291 -0.565826
C -2.274867 -0.825811 -0.215789
N -2.078514 0.554126 -0.094690
C -0.873891 1.163547 -0.319676
C 0.185779 0.371923 -0.658255
N 1.488561 0.687140 -0.877945
C 2.096089 -0.443196 -1.174203
C 1.195216 -1.601491 -1.142990
O 1.398894 -2.806434 -1.342219
N -0.003864 -1.020884 -0.816077
C 3.563126 -0.551564 -1.451519
C 4.344761 -0.872686 -0.189530
C 4.299008 -2.157953 0.359610
C 4.990606 -2.451069 1.531235
C 5.736991 -1.462242 2.171069
C 5.783880 -0.179026 1.632393
C 5.089383 0.113008 0.459644
H 5.128726 1.116732 0.043841
H 6.362411 0.597858 2.123875
H 6.278565 -1.691526 3.084022
H 4.947873 -3.454139 1.945951
H 3.704422 -2.921826 -0.134709
H 3.914142 0.390780 -1.881428
H 3.720581 -1.342190 -2.193379
C -0.802017 2.625695 -0.149304
C -0.033663 3.410501 -1.017100
C 0.012972 4.789550 -0.849917
C -0.705731 5.397627 0.177697
C -1.472426 4.621073 1.043314

C -1.522779 3.240928 0.881964
H -2.102306 2.639319 1.576511
H -2.027976 5.088243 1.850073
H -0.668085 6.475172 0.303555
H 0.607031 5.392107 -1.529647
H 0.518391 2.936938 -1.820571
C -3.625144 -1.340680 0.085522
C -4.383632 -0.784464 1.122693
C -5.651625 -1.280263 1.409172
C -6.173907 -2.338548 0.669485
C -5.423613 -2.897000 -0.363442
C -4.160547 -2.397027 -0.660181
H -3.592848 -2.813550 -1.486786
H -5.828268 -3.715400 -0.950612
H -7.163214 -2.724497 0.894544
H -6.227991 -0.843023 2.218416
H -3.975108 0.021946 1.725709
H -1.283976 -2.686201 -0.632411
H -2.899222 1.141918 -0.044011

3H
0 1
C -0.771889 -1.539748 -0.883525
C -1.730387 -0.807320 -0.229650
N -1.394676 0.507518 0.026432
C -0.246607 1.105779 -0.250831
C 0.753904 0.301380 -0.934292
N 1.967460 0.664958 -1.207876
C 2.596810 -0.451221 -1.860459
C 1.727303 -1.765063 -1.788091
O 1.883352 -2.860458 -1.233136
N 0.369509 -0.973091 -1.267304
C 4.087228 -0.549111 -1.586230
C 4.434791 -0.427423 -0.119832
C 3.957503 -1.360514 0.807681
C 4.270366 -1.238702 2.158371
C 5.068299 -0.185822 2.604541
C 5.548050 0.746444 1.688644
C 5.229877 0.625056 0.337068
H 5.604057 1.360106 -0.371391
H 6.169292 1.571864 2.024424
H 5.312870 -0.093009 3.658675
H 3.890036 -1.969483 2.866755
H 3.325302 -2.173938 0.459194
H 4.581947 0.245855 -2.155261
H 4.439626 -1.505084 -1.990371
C -0.037967 2.497728 0.150696
C 0.692959 3.364239 -0.674481
C 0.846626 4.695978 -0.312005
C 0.291857 5.170057 0.875188
C -0.419792 4.308336 1.707452
C -0.587098 2.977132 1.349577
H -1.107855 2.307827 2.028768
H -0.836307 4.669295 2.641757
H 0.421690 6.210069 1.156993
H 1.403197 5.365915 -0.958920
H 1.131898 2.990583 -1.591172
C -3.048386 -1.301716 0.198993
C -3.596880 -0.910228 1.426540
C -4.843025 -1.385937 1.820347
C -5.545652 -2.268642 1.003825
C -4.999507 -2.670578 -0.213411

C -3.762021 -2.184702 -0.620034
H -3.357028 -2.475274 -1.584788
H -5.544671 -3.353651 -0.856899
H -6.515575 -2.643012 1.315366
H -5.257201 -1.076404 2.774494
H -3.041720 -0.257888 2.095410
H -0.892678 -2.594266 -1.107106
O 2.330874 -0.439185 -3.289419
O 1.641045 -1.729585 -3.244741
H -2.120251 1.084269 0.447122

2′
-1 1
C 1.45911 -0.74586 -0.72538
N 0.08997 -0.59510 -0.45079
C -0.87057 -1.57230 -0.44384
C -2.15690 -1.20439 -0.17602
N -2.48067 0.13109 0.01658
C -1.59414 1.09019 -0.00256
C -0.16917 0.77654 -0.22257
N 0.87094 1.53474 -0.25950
C 2.04575 0.66351 -0.50829
C -2.06544 2.47792 0.21974
C -1.26959 3.60425 -0.06195
C -1.77144 4.89123 0.13979
C -3.06483 5.07970 0.62889
C -3.86275 3.96607 0.91613
C -3.37069 2.68143 0.71241
C -3.27289 -2.17469 -0.10612
C -4.59582 -1.74127 -0.30121
C -5.65631 -2.64573 -0.25285
C -5.41869 -4.00034 -0.00649
C -4.10809 -4.44108 0.19886
C -3.04687 -3.53809 0.15575
O 1.98934 -1.79437 -1.03227
H -0.52300 -2.57342 -0.66497
H -0.26184 3.46489 -0.43120
H -1.14489 5.74948 -0.08729
H -3.44931 6.08331 0.78892
H -4.86915 4.10168 1.30268
H -3.98581 1.81677 0.93524
H -4.78131 -0.69020 -0.49402
H -6.67125 -2.29061 -0.41062
H -6.24539 -4.70415 0.03301
H -3.91126 -5.48929 0.40677
H -2.04013 -3.89580 0.35137
C 2.92061 0.69040 0.78037
H 2.28284 0.47228 1.64426
H 3.24371 1.73321 0.82974
C 4.11059 -0.23293 0.73929
C 4.19296 -1.34777 1.58336
C 5.16121 0.02006 -0.15870
C 5.30348 -2.19571 1.54293
H 3.38175 -1.55420 2.27890
C 6.26986 -0.82693 -0.19815
H 5.06175 0.89090 -0.80710
C 6.34652 -1.93746 0.65014
H 5.35279 -3.05541 2.20685
H 7.08050 -0.62319 -0.89473
H 7.21151 -2.59536 0.61541
O 2.74532 0.99739 -1.66575
O 3.49177 2.25242 -1.47303

<u>Additional references</u>:

42. Ando, Y. *et al.* Development of a quantitative bio/chemiluminescence spectrometer determining quantum yields: Re-examination of the aqueous luminol chemiluminescence standard. *Photochem. Photobiol.* **83**, 1205–1210 (2007).
43. Li, W. & Godzik, A. Cd-hit: a fast program for clustering and comparing large sets of protein or nucleotide sequences. *Bioinformatics* **22**, 1658–1659 (2006).
44. Farrell, D. P. *et al.* Deep learning enables the atomic structure determination of the Fanconi Anemia core complex from cryoEM. *IUCrJ* **7**, 881–892 (2020).
45. Zhang, Y. & Skolnick, J. TM-align: a protein structure alignment algorithm based on the TM-score. *Nucleic Acids Res.* **33**, 2302–2309 (2005).
46. Needleman, S. B. & Wunsch, C. D. A general method applicable to the search for similarities in the amino acid sequence of two proteins. *J. Mol. Biol.* **48**, 443–453 (1970).
47. Oda, T., Lim, K. & Tomii, K. Simple adjustment of the sequence weight algorithm remarkably enhances PSI-BLAST performance. *BMC Bioinformatics* **18**, 288 (2017).
48. Altschul, S. F. *et al.* Gapped BLAST and PSI-BLAST: a new generation of protein database search programs. *Nucleic Acids Res.* **25**, 3389–3402 (1997).
49. Coventry, B. & Baker, D. Protein sequence optimization with a pairwise decomposable penalty for buried unsatisfied hydrogen bonds. *PLoS Comput. Biol.* **17**, (2021).
50. Smith, A. J. T. *et al.* Structural Reorganization and Preorganization in Enzyme Active Sites: Comparisons of Experimental and Theoretically Ideal Active Site Geometries in the Multistep Serine Esterase Reaction Cycle. *J. Am. Chem. Soc.* **130**, 15361–15373 (2008).
51. Salomon-Ferrer, R., Case, D. A. & Walker, R. C. An overview of the Amber biomolecular simulation package. *Wiley Interdiscip. Rev. Comput. Mol. Sci.* **3**, 198–210 (2013).
52. Kellogg, E. H., Leaver-Fay, A. & Baker, D. Role of conformational sampling in computing mutation-induced changes in protein structure and stability. *Proteins* **79**, 830–838 (2011).
53. Park, H. *et al.* Simultaneous optimization of biomolecular energy functions on features from small molecules and macromolecules. *J. Chem. Theory Comput.* **12**, 6201–6212 (2016).
54. Klein, J. C. *et al.* Multiplex pairwise assembly of array-derived DNA oligonucleotides. *Nucleic Acids Res.* **44**, (2016).
55. Loening, A. M., Wu, A. M. & Gambhir, S. S. Red-shifted Renilla reniformis luciferase variants for imaging in living subjects. *Nat. Methods* **4**, 641–643 (2007).
56. Liang, J., Feng, X., Hait, D. & Head-Gordon, M. Revisiting the performance of time-dependent density functional theory for electronic excitations: Assessment of 43 popular and recently developed functionals from rungs one to four. *J. Chem. Theory Comput.* **18**, 3460–3473 (2022).
57. Chai, J.-D. & Head-Gordon, M. Long-range corrected hybrid density functionals with damped atom-atom dispersion corrections. *Phys. Chem. Chem. Phys.* **10**, 6615–6620 (2008).
58. Ditchfield, R., Hehre, W. J. & Pople, J. A. Self-consistent molecular-orbital methods. IX. An extended Gaussian-type basis for molecular-orbital studies of organic molecules. *J. Chem. Phys.* **54**, 724–728 (1971).

59. Grimme, S. Exploration of chemical compound, conformer, and reaction space with meta-dynamics simulations based on tight-binding quantum chemical calculations. *J. Chem. Theory Comput.* **15**, 2847–2862 (2019).
60. Pracht, P., Bohle, F. & Grimme, S. Automated exploration of the low-energy chemical space with fast quantum chemical methods. *Phys. Chem. Chem. Phys.* **22**, 7169–7192 (2020).
61. Luchini, G., Alegre-Requena, J. V., Funes-Ardoiz, I. & Paton, R. S. GoodVibes: automated thermochemistry for heterogeneous computational chemistry data. *F1000Res.* **9**, 291 (2020).
62. Li, Y.-P., Gomes, J., Mallikarjun Sharada, S., Bell, A. T. & Head-Gordon, M. Improved force-field parameters for QM/MM simulations of the energies of adsorption for molecules in zeolites and a free rotor correction to the rigid rotor harmonic oscillator model for adsorption enthalpies. *J. Phys. Chem. C Nanomater. Interfaces* **119**, 1840–1850 (2015).
63. Götz, A. W. *et al.* Routine microsecond molecular dynamics simulations with AMBER on GPUs. 1. Generalized Born. *J. Chem. Theory Comput.* **8**, 1542–1555 (2012).
64. Becke, A. D. Density-functional thermochemistry. III. The role of exact exchange. *J. Chem. Phys.* **98**, 5648–5652 (1993).
65. Grimme, S., Antony, J., Ehrlich, S. & Krieg, H. A consistent and accurate ab initio parametrization of density functional dispersion correction (DFT-D) for the 94 elements H-Pu. *J. Chem. Phys.* **132**, 154104 (2010).
66. Grimme, S., Ehrlich, S. & Goerigk, L. Effect of the damping function in dispersion corrected density functional theory. *J. Comput. Chem.* **32**, 1456–1465 (2011).
67. Meiler, J. & Baker, D. ROSETTALIGAND: Protein-small molecule docking with full side-chain flexibility. *Proteins* **65**, 538–548 (2006).
68. Davis, I. W. & Baker, D. RosettaLigand docking with full ligand and receptor flexibility. *J. Mol. Biol.* **385**, 381–392 (2009).
69. Davis, I. W., Raha, K., Head, M. S. & Baker, D. Blind docking of pharmaceutically relevant compounds using RosettaLigand. *Protein Sci.* **18**, 1998–2002 (2009).
70. Wang, J., Wolf, R. M., Caldwell, J. W., Kollman, P. A. & Case, D. A. Development and testing of a general amber force field. *J. Comput. Chem.* **25**, 1157–1174 (2004).
71. Bayly, C. I., Cieplak, P., Cornell, W. & Kollman, P. A. A well-behaved electrostatic potential based method using charge restraints for deriving atomic charges: the RESP model. *J. Phys. Chem.* **97**, 10269–10280 (1993).
72. Besler, B. H., Merz, K. M. & Kollman, P. A. Atomic charges derived from semiempirical methods. *J. Comput. Chem.* **11**, 431–439 (1990).
73. Singh, U. C. & Kollman, P. A. An approach to computing electrostatic charges for molecules. *J. Comput. Chem.* **5**, 129–145 (1984).
74. Jorgensen, W. L., Chandrasekhar, J., Madura, J. D., Impey, R. W. & Klein, M. L. Comparison of simple potential functions for simulating liquid water. *J. Chem. Phys.* **79**, 926–935 (1983).
75. Maier, J. A. *et al.* Ff14SB: Improving the accuracy of protein side chain and backbone parameters from ff99SB. *J. Chem. Theory Comput.* **11**, 3696–3713 (2015).
76. Darden, T., York, D. & Pedersen, L. Particle mesh Ewald: An$N \cdot \log(N)$ method for Ewald sums in large systems. *J. Chem. Phys.* **98**, 10089–10092 (1993).

77. Roe, D. R. & Cheatham, T. E., III. PTRAJ and CPPTRAJ: Software for processing and analysis of molecular dynamics trajectory data. *J. Chem. Theory Comput.* **9**, 3084–3095 (2013).
78. Loening, A. M., Dragulescu-Andrasi, A. & Gambhir, S. S. A red-shifted Renilla luciferase for transient reporter-gene expression. *Nat. Methods* **7**, 5–6 (2010).
79. Dijkema, F. M. *et al.* Flash properties of Gaussia luciferase are the result of covalent inhibition after a limited number of cycles. *Protein Sci.* **30**, 638–649 (2021).
80. Schenkmayerova, A. *et al.* Engineering the protein dynamics of an ancestral luciferase. *Nat. Commun.* **12**, (2021).
