# Supplementary material for: De novo design of luciferases using deep learning
Source: Nature. 2023 Feb 22;614(7949):774–80. doi: 10.1038/s41586-023-05696-3 (PMC9946828; doi:10.1038/s41586-023-05696-3)
Supplement: Supplementary file 2 — Reporting Summary [file 41586_2023_5696_MOESM2_ESM.pdf]

nature portfolio

Corresponding author(s): Andy Hsien-Wei Yeh and David Baker

Last updated by author(s): Nov 15, 2022

Reporting Summary

Nature Portfolio wishes to improve the reproducibility of the work that we publish. This form provides structure for consistency and transparency in reporting. For further information on Nature Portfolio policies, see our Editorial Policies and the Editorial Policy Checklist.

Statistics

For all statistical analyses, confirm that the following items are present in the figure legend, table legend, main text, or Methods section.

n/a	Confirmed	
☐	☒	The exact sample size (*n*) for each experimental group/condition, given as a discrete number and unit of measurement
☐	☒	A statement on whether measurements were taken from distinct samples or whether the same sample was measured repeatedly
☒	☐	The statistical test(s) used AND whether they are one- or two-sided<br>*Only common tests should be described solely by name; describe more complex techniques in the Methods section.*
☒	☐	A description of all covariates tested
☒	☐	A description of any assumptions or corrections, such as tests of normality and adjustment for multiple comparisons
☐	☒	A full description of the statistical parameters including central tendency (e.g. means) or other basic estimates (e.g. regression coefficient) AND variation (e.g. standard deviation) or associated estimates of uncertainty (e.g. confidence intervals)
☒	☐	For null hypothesis testing, the test statistic (e.g. *F*, *t*, *r*) with confidence intervals, effect sizes, degrees of freedom and *P* value noted<br>*Give P values as exact values whenever suitable.*
☒	☐	For Bayesian analysis, information on the choice of priors and Markov chain Monte Carlo settings
☒	☐	For hierarchical and complex designs, identification of the appropriate level for tests and full reporting of outcomes
☒	☐	Estimates of effect sizes (e.g. Cohen's *d*, Pearson's *r*), indicating how they were calculated

Our web collection on statistics for biologists contains articles on many of the points above.

Software and code

Policy information about availability of computer code

Data collection	The Rosetta macromolecular modelling suite (https://www.rosettacommons.org) is freely available to academic and non-commercial users. Commercial licences for the suite are available through the University of Washington Technology Transfer Office.<br><br>The source code for RIF docking implementation is freely available at https://github.com/rifdock/rifdock.<br><br>All relevant scripts and an accompanying Jupiter notebook for family-wide hallucination scaffold generation are available here: https://files.ipd.uw.edu/pub/luxSit/scaffold_generation.tar.gz<br><br>All generated scaffolds are available here: https://files.ipd.uw.edu/pub/luxSit/scaffolds.tar.gz<br><br>Design scripts for active LuxSit are available here: https://files.ipd.uw.edu/pub/luxSit/luciferase_designs_methods.zip
Data analysis	The molecular mass of each protein was deconvoluted by Bioconfirm software (10.0) using a total entropy algorithm. Microscopic fluorescence and luminescence images were analyzed using NIS Elements 5.30 software. Data were analyzed and plotted using GraphPad Prism 8, Python 3.8, DNAWorks2.0, seaborn0.12.1, and matplotlib3.6.2.

For manuscripts utilizing custom algorithms or software that are central to the research but not yet described in published literature, software must be made available to editors and reviewers. We strongly encourage code deposition in a community repository (e.g. GitHub). See the Nature Portfolio guidelines for submitting code & software for further information.

Data

Policy information about availability of data

All manuscripts must include a data availability statement. This statement should provide the following information, where applicable:

- Accession codes, unique identifiers, or web links for publicly available datasets
- A description of any restrictions on data availability
- For clinical datasets or third party data, please ensure that the statement adheres to our policy

Source data for Fig. 2, 3, and 4 are available online. The gene sequence for LuxSit-i has been deposited to GenBank under the accession number OP820699. Design models of LuxSit and LuxSit-i are available online in LuxSit_models.zip. Codon-optimized plasmids encoding LuxSit-i for bacterial and mammalian expression are available through Addgene.

Human research participants

Policy information about studies involving human research participants and Sex and Gender in Research.

Reporting on sex and gender	N/A
Population characteristics	N/A
Recruitment	N/A
Ethics oversight	N/A

Note that full information on the approval of the study protocol must also be provided in the manuscript.

Field-specific reporting

Please select the one below that is the best fit for your research. If you are not sure, read the appropriate sections before making your selection.

☒ Life sciences ☐ Behavioural & social sciences ☐ Ecological, evolutionary & environmental sciences

For a reference copy of the document with all sections, see nature.com/documents/nr-reporting-summary-flat.pdf

Life sciences study design

All studies must disclose on these points even when the disclosure is negative.

Sample size	No statistical methods were used to pre-determine the sample size. The number of designs we ordered is limited by the availability of adaptor pairs and the size of Oligo pool.
Data exclusions	No sample was excluded from the data analysis.
Replication	Results were reproduced using different batches of pure proteins on different days. Coomassie-stained SDS PAGE gels were done at least twice for each experiment. Microscopic fluorescence and luminescence images were repeated in biological triplicate with similar results.
Randomization	For the cellular imaging and assay, cells were randomly separated into experimental groups.
Blinding	Researchers were not blinded to the experiments described in this study.

Reporting for specific materials, systems and methods

We require information from authors about some types of materials, experimental systems and methods used in many studies. Here, indicate whether each material, system or method listed is relevant to your study. If you are not sure if a list item applies to your research, read the appropriate section before selecting a response.

Materials & experimental systems

n/a	Involved in the study
☒	☐ Antibodies
☐	☒ Eukaryotic cell lines
☒	☐ Palaeontology and archaeology
☒	☐ Animals and other organisms
☒	☐ Clinical data
☒	☐ Dual use research of concern

Methods

n/a	Involved in the study
☒	☐ ChIP-seq
☒	☐ Flow cytometry
☒	☐ MRI-based neuroimaging

Eukaryotic cell lines

Policy information about cell lines and Sex and Gender in Research

Cell line source(s)	HEK293T (ATCC, CRL-11268) and HeLa (ATCC, CCL-2)
Authentication	Autheticated by vendors. Cells were not authenticated in the lab
Mycoplasma contamination	All cells were tested negative for Mycoplasma by the provider, and it was not further confirmed in the lab.
Commonly misidentified lines (See ICLAC register)	no commonly misidentified cell lines were used in the study
